# Self-other overlap: A unique predictor of willingness to work with people with disability as part of one’s career

**DOI:** 10.1371/journal.pone.0220722

**Published:** 2019-08-12

**Authors:** Michael Ioerger, Laura V. Machia, Margaret A. Turk

**Affiliations:** 1 Department of Psychology, Syracuse University, Syracuse, New York, United States of America; 2 Department of Physical Medicine & Rehabilitation, State University of New York Upstate Medical University, Syracuse, New York, United States of America; Middlesex University, UNITED KINGDOM

## Abstract

**Background:**

People with disability (PWD) often rely on others, both for direct support and for the creation of enabling environments to meet their needs. This need makes it crucial for professionals to be willing to work with PWD, and for people to pursue careers that focus on supporting PWD.

**Objectives:**

To explore self-other overlap as a unique predictor of willingness to work with PWD as part of one’s career, using three studies.

**Methods:**

Studies 1 and 2 used cross-sectional surveys of college undergraduates to explore: 1. whether an association between self-other overlap and willingness to work with PWD exists, and 2. whether self-other overlap is a unique predictor, controlling for attitudes and empathy. Study 3 investigated whether self-other overlap is associated with the groups with whom the students indicated they want (and do not want) to work as part of their career.

**Results:**

Across the three studies, self-other overlap was uniquely associated with students’ willingness to work with PWD as part of one’s profession, even when controlling for attitudes and empathy.

**Conclusions:**

Self-other overlap may be an important additional factor to take into consideration when developing interventions targeted toward promoting working with PWD.

## Introduction

People with disability (PWD) are considered disabled because some aspect of their self functions in a way that impacts their ability to care for themselves or fully participate in society in the same way as others [1]. Given their different abilities, and, in some cases, their need for assistance or accommodation, PWD often rely on others, both for direct support and for the creation of enabling environments to meet their needs [2]. For children, support may come largely from parents and other family members, but as PWD move through the educational system and into adulthood, this support and assistance often needs to come from other adults as either a focus of, or as part of, their professional work (e.g., physical therapists, interpreters, personal care assistants, medical office receptionists, architects) [2]. This need makes it crucial for professionals to be willing to work with PWD, and for people to pursue careers that focus on supporting PWD. However, it can be challenging to find people who are willing to work with this population.

Working with PWD is often characterized as intimidating and challenging because it can take time, effort, and a willingness to work with people to overcome unique challenges [3]. Additionally, many people feel uncomfortable working with PWD because they seem so different from themselves [4,5]. The combination of PWD being seen as both costly with whom to work and fundamentally different from themselves creates substantial barriers to motivating people to work with this portion of the population [2]. However, choosing to work with PWD as part of one’s career is a type of helping behavior, and considering it as such might provide greater insight into how to facilitate people’s interest in working with PWD.

### Factors underlying long-term helping behavior

Helping PWD can take both short-term and long-term forms. For example, while out shopping, a physician may be asked by a person with disability to make a donation to a non-profit organization that serves him and other PWD. Making this donation would constitute short-term helping. However, the same physician might devote her career to serving patients with disability. This would be a long-term form of helping.

The factors underlying the expression of helping behavior are influenced by the magnitude of the investment and the time scale [6,7]. In any given moment, empathy, emotion, perceptions, social pressure, and moral preferences are likely the best predictors of helping behavior, especially when helping can be completed in a relatively short time-frame with one discrete action [7–9]. Because of this, interventions to reduce the prejudice experienced by PWD are often evaluated based on their ability to change individuals’ reported attitudes or levels of empathy [10–13]. However, when helping requires a larger investment over a longer period of time with repeated actions, selfish interests become much stronger predictors of helping behavior [7]. For example, people are more likely to provide greater amounts of help over a longer period of time when they are helping those who are close to them, rather than strangers [7,14–16]. Additionally, research into the antecedents of volunteerism has found that self-serving motives and a psychological sense of community are associated with providing this kind of long-term helping [7,17–22]. Because of the influence of these selfish interests, self-other overlap may be a key factor in directing helping behavior, especially with respect to helping behavior directed towards apparent outgroup members [15,16].

### Self-other overlap & long-term helping

Closeness arises when people subjectively feel connected to someone, as well as when they have great frequency and diversity of contact with someone. In other words, it is not just influenced by thoughts and feelings related to the target, but also by interactions [23,24]. This sense of closeness can be assessed by evaluating self-other overlap, a measure of how much overlap an individual sees between him- or herself and another individual or group. Higher levels of overlap represent feeling a greater sense of closeness and connection with the target. People who perceive a high degree of self-other overlap with another are likely to treat the other like they would want to be treated, and are more likely to expend substantial resources helping [6,25].

The self-other overlap scale [26,27] uses a visual representation of closeness to capture the way people construe themselves with respect to a target using progressively overlapping circles. The more the circles representing the self and the other overlap, the greater the feelings of closeness between themselves and the target [26,27]. It has been shown that reporting high levels of self-other overlap with a target using this measure captures a combination of both subjectively feeling close and objectively being close (i.e., frequency of contact, diversity of contact) [27]. Thus, self-other overlap is not just influenced by thoughts and feelings related to the target group, but also by intergroup contact [23,24].

Mechanistically, the people with whom individuals feel the greatest overlap are seen and treated more like the individuals treat themselves than people with whom they see and feel less overlap [26–28]. This sense of closeness then has an impact on individuals’ willingness to help the target, such that the people to whom individuals feel the closest and see most like themselves (e.g., friends, family members, neighbors) are the ones they are most likely to help in an investment-heavy way [6,25]. There are several explanations for why people help those with whom they share self-other overlap. Evolutionary psychologists suggest that this process is driven by a biological desire to ensure the survival of genetic material through kin selection [29–31]. The underlying logic being that, historically, the ones to whom individuals felt the closest, and with whom they had the most frequent, diverse, and emotionally intimate relationships would have been family members. Thus, this mechanism would have directed long-term helping behavior and investment toward the people who are most genetically similar to ourselves. Other psychological theorists suggest this association between overlap and helping is driven by an expectation of reciprocity, with people who feel closer to us or who seem more like us, being more likely to help us in the future than people who feel less close to us or are less like us [14,32]. Regardless of the basis for this mechanism, there is clear empirical evidence that people feel most driven to support others to whom they feel close and are most inclined to treat them like themselves, especially when helping requires a long-term investment [6,25].

### Connecting self-other overlap to working with people with disability

One key way that working with people with disability is different than working with other groups of people (e.g., females, adults 25–35 years of age, school teachers) is that, on average, providing a service for people with disability requires planning and effort that is greater than most other groups of people. Therefore, making a conscious choice to work with this group is not just about thinking positively about them, knowing their needs, and having the skills to meet them. It is also about being willing to invest putting in more to the interaction and getting out less, at least in terms of monetary reimbursement, in return. This component of the dynamic suggests there is at least a part of this choice that is motivated by prosocial thoughts.

Conceptualizing working with people with disability as a long-term prosocial helping behavior provides a frame that can be used to evaluate the potential limitations of techniques employed by interventions to promote working with people with disability. For example, a curriculum change was implemented at a medical school in an attempt to enhance students’ attitudes and skills related to working with patients with disability [33]. This curriculum change included many elements intended to reduce prejudice and change behavior, including: integrating the changes across all four years of medical school, raising awareness of disability-related issues, having small-group interactions with people with disability, and having clinical skill practice sessions include people with disability [33]. However, all of these experiences and interactions were couched in the context of *learning about people with disability*, emphasizing the difference between the medical students and people with disability, and the difference between people with disability and the rest of their patients. Viewing this study from the perspective of self-other overlap helps to reinforce the authors’ assertion that some students ended-up having more negative feelings towards patients with disability after the intervention because patients with disability were seen as being even more different than other patients [33]. Thus, while this intervention may have been well suited to address attitudes, knowledge, and skills, it was missing elements that would have enhanced feelings of closeness between the medical students and people with disability.

Interventions that have demonstrated the most success in recruiting professionals to work with people with disability incorporate activities that increase individuals’ sense of closeness [5,34,35]. These activities include facilitating social interactions with people with disability outside of the work context, or extended periods of time working with PWD [33,36–39]. The success of these interventions is often attributed to changes in participants’ attitudes toward people with disability or feelings of empathy. However, interventions targeting attitudes and empathy have not had consistently positive results [33]. Thus, overlooking the contribution of influencing closeness may result in the literature appearing more inconsistent than it actually may be.

### The current work

The current work explored the association between self-other overlap and willingness to work with PWD in three studies. Self-other overlap was selected as the target for investigation because it utilizes a visual analogue scale consisting of seven pairs of circles progressively increasing in their degree of overlap [26–28] to provide a simple and efficient method for capturing a complex sense of a person’s overall perception of closeness, similarity, and integration with respect to a group of people [40–42]. Additionally, this particular visual scale has empirically been shown to be uniquely associated with expressing long-term helping behavior toward targets identified as having high levels of overlap with the self [6,25]. Study 1 provided an initial test of the association between self-other overlap and willingness to work with PWD. Study 2 provided an opportunity to replicate the findings of study 1 and investigate whether self-other overlap is a unique predictor of willingness to work with PWD, controlling for attitudes and empathy. Finally, study 3 investigated whether the association between self-other overlap and willingness to work with PWD is generalizable to other groups of people. It was hypothesized that, across all three studies, self-other overlap would be a unique, positive predictor of willingness to work with PWD as part of their future career.

## Study 1

Study 1 provided an initial test of the study hypothesis by evaluating whether an association exists between ratings of self-other overlap and willingness to work with PWD. Willingness to work with PWD was operationalized in two ways: 1. the extent to which a person is open to working with PWD as part of one’s future career, and 2. the likelihood of working primarily with PWD as part of one’s future career. Self-other overlap was predicted to be positively associated with both measures of willingness to work with PWD as part of one’s future career. It was anticipated that these associations would exist even after controlling for age and sex. The contributions of age and sex were evaluated because previous studies have found that women and people who are older have more positive attitudes toward PWD [43–45].

### Materials and methods

#### Procedure

For this study, participants completed 6 brief survey questions near the end of two social psychology studies investigating other constructs. The Syracuse University Institutional Review Board approved each of these studies. The survey questions captured demographic information, self-other overlap with PWD, openness to working with PWD, and likelihood of working primarily with PWD.

#### Participants

Participants were undergraduates recruited from the psychology department participant pool who chose to complete the study items. The overall study sample was created by combining the responses of the samples obtained from the two studies (A & B) that integrated these questions into their surveys.

An a priori power analysis was conducted using G*Power 3.1. F tests were specified as the test family, and the statistical test was specified as linear multiple regression (fixed model, R^2^ increase). The input parameters were: partial R^2^ = .05 (effect size f^2^ = .05263), α-error probability = .05, Power (1 –β-error probability) = .80, number of tested predictors = 3. This analysis determined 220 participants would provide sufficient power (power = 0.80) to detect the effect of a predictor with a partial R^2^ of .05.

#### Measures

**Fig 1:**
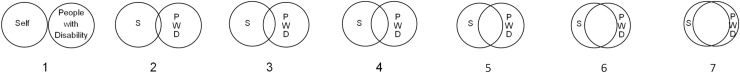
Self-other overlap with people with disability scale. Participants were asked to: Please select the pair of circles that best represents your relationship with people with disability. [S = Self, PWD = People with Disability].

**Self-other overlap with people with disability.** The Inclusion of Other in the Self Scale [27] was modified to assess participants’ self-reported ratings of self-other overlap with PWD. Participants were asked to select the pair of circles that best represents their relationship with PWD. Each of the 7 pairs of circles represented different levels of overlap between the self and PWD (Fig 1), with circles representing higher levels of overlap being associated with higher scale values (1 = two non-overlapping circles, 7 = two almost completely overlapping circles). The modifications made to the original Inclusion of Other in the Self Scale [27] item were based on similar changes to the label of the target used in previous work to assess self-other overlap both with individuals [25,46] and groups [40,41].

**Openness to working with people with disability.** Openness to working with PWD was assessed by asking participants to use a slider to indicate their extent of openness to working with PWD as part of their future career. The slider allowed participants to provide their response on a 101-point scale (0 = *Not at All Open*, 100 = *Very Open*).

**Likelihood of working primarily with people with disability.** Likelihood of working primarily with PWD was assessed by asking participants to use a slider to indicate the extent to which they are likely to work primarily with PWD as part of their future career. The slider allowed participants to provide their response on a 101-point scale (0 = *Not at All Likely*, 100 = *Very Likely*).

**Age.**Age was assessed by allowing participants to type a number into a textbox.

**Level in college.** Level in college was assessed by allowing participants to select one of 5 options (1 = *Freshman*, 2 = *Sophomore*, 3 = *Junior*, 4 = *Senior*, 5 = *Other*). Participants who selected “Other” were asked to provide more information in a textbox.

**Sex.** Sex was assessed by allowing participants to select either male (0) or female (1).

### Study 1 results

#### Overall study descriptive statistics

Overall, there were 624 participants who completed the study items. The overall sample was 70.5% freshman (n = 440; 19.2% sophomore, 4.3% junior, 5.4% senior, and .5% other) and 63.5% female (n = 396), with ages ranging from 18–30 (*M* = 18.71, *SD* = 1.29). Study A provided a total of 212 participants who were 72.6% freshman (n = 154; 16.0% sophomore, 4.2% junior, 6.6% senior, and .5% other) and 63.7% female (n = 135; 36.3% male), with an age range of 18–30 (M = 18.85, SD = 1.54). Study B provided a total of 412 participants who were 69.4% freshman (n = 286; 20.9% sophomore, 4.4% junior, 4.9% senior, and .5% other) and 63.3% female (n = 261; 36.7% male), with an age range of 18–30 (M = 18.64, SD = 1.13). Based on the obtained sample size and the a priori power calculations, this study was deemed to be sufficiently powered to test for the hypothesized associations.

Table 1 provides the means and standard deviations for participants’ ratings of self-other overlap, openness to working with PWD, and likelihood of working primary with PWD as part of their career. The mean for likelihood of primarily working with PWD (*M* = 37.53, *SD* = 28.72) is 28 points lower than the mean for openness to working with PWD (*M =* 65.56, *SD* = 28.39). Differences between the means for samples A and B were tested using independent samples *t*-tests. There was a statistically significant difference (*p* = .05) between sample A (*M* = 68.69, *SD* = 28.23) and sample B (*M* = 63.97, *SD* = 28.37) with respect to ratings of openness to working with PWD. However, the overall pattern of the findings of the following analyses do not change if they are run separately with each sample.

**Table 1:** Overall study and sample descriptive statistics for Study 1.

	Overall M (SD)	Sample A M (SD) / %	Sample B M (SD) / %	*t*	*p*
Self-Other Overlap	3.45 (1.60)	3.35 (1.60)	3.50 (1.59)	*t* (1, 621) = -1.07	.29
Openness	65.56 (28.39)	68.69 (28.23)	63.97 (28.37)	*t* (1, 620) = 1.97	.05
Primary	37.53 (28.72)	38.69 (29.81)	36.94 (28.17)	*t* (1, 620) = .717	.47

*Note*. Self-Other: 1 = very little self-other overlap (i.e., the circles in the image are just touching), 7 = quite a bit of self-other overlap (i.e., the circles in the image are almost completely overlapping). Openness = Openness to working with people with disability: 0 = *Not at All Open*, 100 = *Very Open*. Primary = Likelihood of working primarily with people with disability: 0 = *Not at All Likely*, 100 = *Very Likely*.

**Fig 2:**
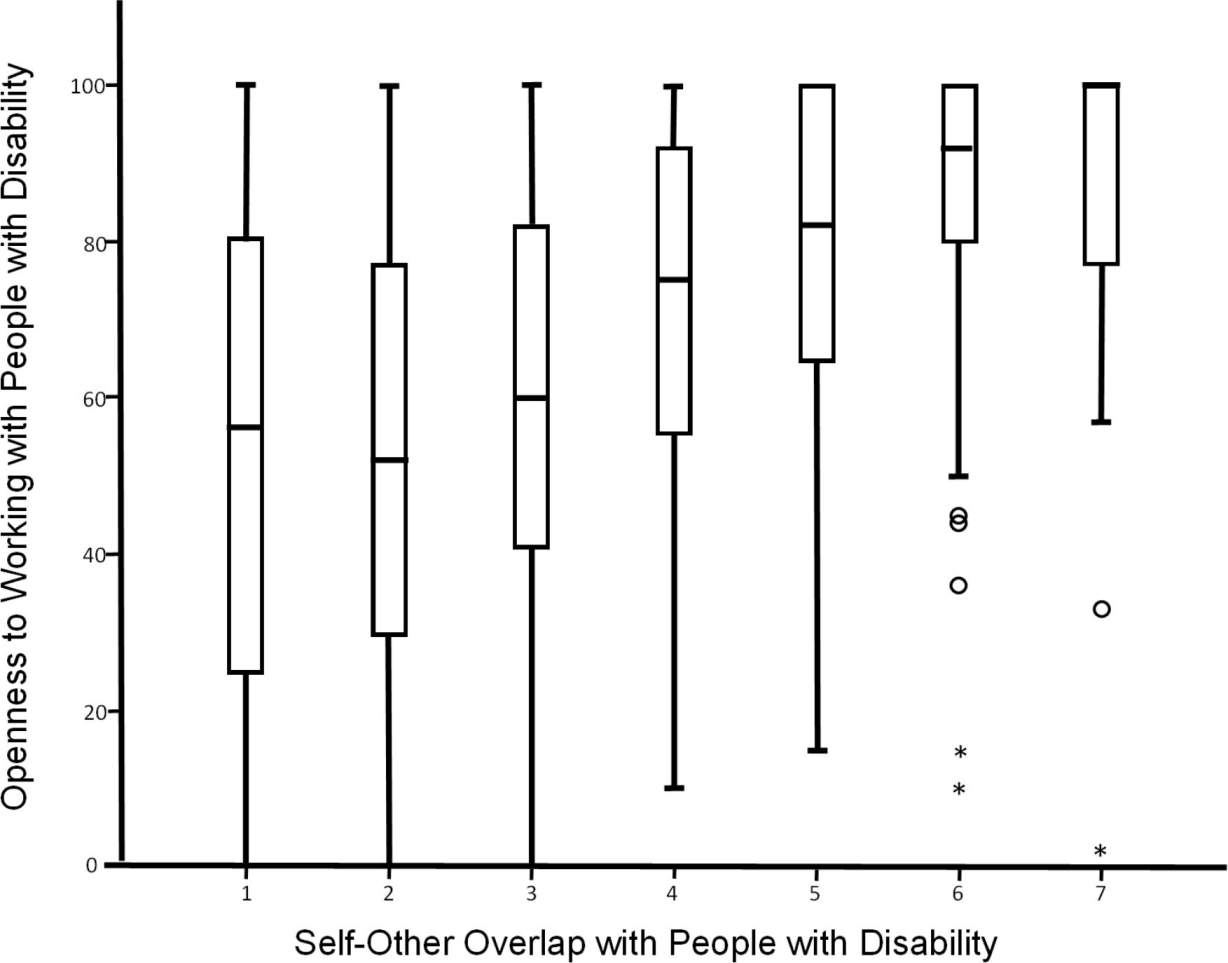
Boxplot of openness to working with people with disability by level of self-other overlap. Openness to working with people with disability: 0 = *Not at All Open*, 100 = *Very Open*. Self-Other: 1 = very little self-other overlap (i.e., the circles in the image are just touching), 7 = quite a bit of self-other overlap (i.e., the circles in the image are almost completely overlapping).

**Fig 3:**
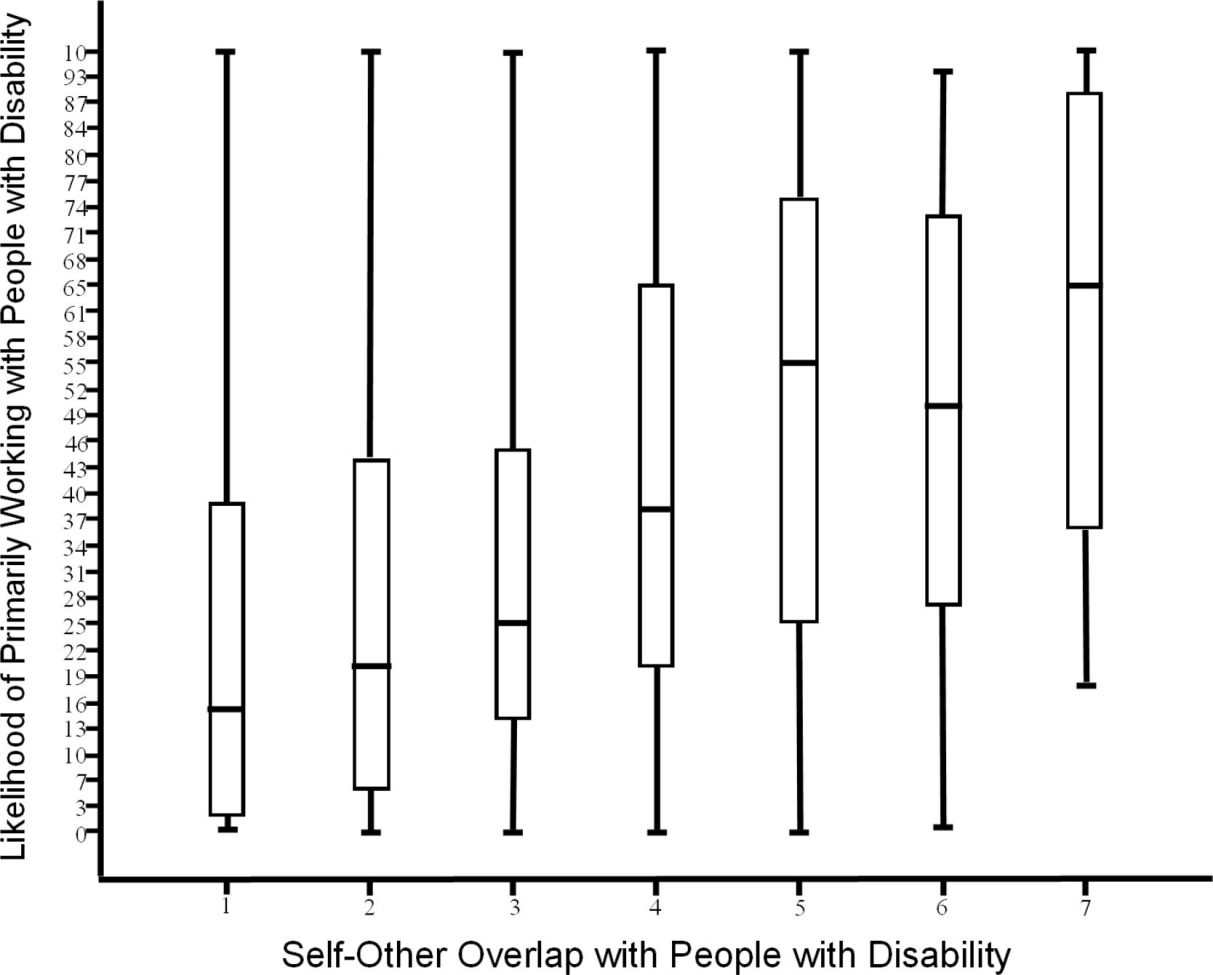
Boxplot of likelihood of working primarily with people with disability by level of self-other overlap. Likelihood of working primarily with people with disability: 0 = *Not at All Likely*, 100 = *Very Likely*. Self-Other: 1 = very little self-other overlap (i.e., the circles in the image are just touching), 7 = quite a bit of self-other overlap (i.e., the circles in the image are almost completely overlapping).

Table 2 provides the bivariate correlations for the study variables. As predicted, there was a positive association between self-other overlap and self-reported openness to working with PWD (*r* = .400, *p* < .05). Additionally, as predicted, there is a positive association between self-other overlap and self-reported likelihood of primarily working with PWD (*r* = .374, *p* < .05). Figs 2 and 3 provide boxplots comparing participants’ responses to either the openness to working with PWD item or the likelihood of working primarily with PWD item, and their responses to the self-other overlap item. These figures highlight the pattern of relationship, with increasing levels of self-other overlap generally associated with higher mean levels of each of the willingness measures.

**Table 2:** Bivariate correlations for Study 1 variables.

	Self-Other Overlap	Openness	Primary	Female	Sex
Self-Other Overlap	-				
Openness	.400*	-			
Primary	.374*	.582*	-		
Sex	-.009	.117*	.080*	-	
Age	.001	.010	.070	-.069	-

*Note*. The asterisks (*) identify significant (*p* < .05) Pearson’s R correlation coefficients. Openness = Openness to Work with People with Disability. Primary = Likelihood of Working Primarily with People with Disability. Sex: 0 = *Male*, 1 = *Female*.

#### Self-other overlap as a predictor of openness to working with people with disability

To further examine the association between self-other overlap and openness to working with PWD, a hierarchical multiple regression analysis was performed (see Table 3). For this analysis, the individual trait factors of sex and age were entered into the first step of the model to evaluate their ability to predict openness to work with PWD. The overall model was significant (*F*(2, 618) = 4.27, *p* = .014), and sex was a significant predictor (*B* = 6.86, *β* = .177, *p* = .004), with females being more open to working with PWD. However, these factors accounted for less than 2% of the variance in openness to working with PWD (*R*^*2*^ = .014).

**Table 3:** Multiple regression analyses for openness to working with people with disability (Study 1).

	B	β	*t*	*p*	R^2^	*F*	df	*p*
Step 1					.014	4.27	2, 618	.014
Sex	6.86	.177	2.91	.004				
Age	.37	.016	.39	.697				
Constant	61.16							
Step 2					.175	43.51	3, 617	< .001
Sex	7.11	.121	3.30	.001				
Age	.24	.010	.27	.784				
Constant	61.02							
Self-Other Overlap	7.13	.401	10.97	< .001				

*Note*. Openness to working with people with disability: 0 = *Not at All Open*, 100 = *Very Open*. Sex: 0 = *Male*, 1 = *Female*. Age (*M* = 18.71) and Self-Other Overlap (*M* = 3.45) in this model were mean-centered.

The second step of the hierarchical multiple regression analysis added self-other overlap to the model, allowing for a test of the association between self-other overlap and openness to working with PWD controlling for the effects of sex and age. This step of analysis revealed self-other overlap was a significant predictor of openness to working with PWD (*B* = 7.13, *β* = .401, *p* < .001), with this overall model predicting 17% of the variance in openness to working with PWD (*R*^*2*^ = .175). Sex remained a significant predictor (*B* = 7.11, *β* = .121, *p* = .001).

#### Self-other overlap as a predictor of primarily working with people with disability

To further examine the association between self-other overlap and likelihood of working primarily with PWD, a hierarchical multiple regression analysis was performed (see Table 4). For this analysis, the individual trait factors of sex and age were entered into the first step of the model to evaluate their ability to predict likelihood of working primarily with PWD. The overall model was significant (*F*(2, 618) = 3.72, *p* = .025), and sex was a significant predictor (*B* = 5.00, *β* = .084, *p* = .037), with females rating themselves as being more likely to primarily work with PWD. However, these factors only account for less than 2% of the variance in likelihood of working primarily with PWD (*R*^*2*^ = .012).

**Table 4:** Multiple regression analyses for likelihood of working primarily with people with disability (Study 1).

	B	β	*t*	*p*	R^2^	*F*	df	*p*
Step 1					.012	3.72	2, 618	.025
Sex	5.00	.084	2.10	.037				
Age	1.69	.076	1.89	.060				
Constant	34.31							
Step 2					.152	36.91	3, 617	< .001
Sex	5.27	.089	2.38	.018				
Age	1.69	.076	2.04	.042				
Constant	34.21							
Self-Other Overlap	6.75	.375	10.10	< .001				

*Note*. Likelihood of working primarily with people with disability: 0 = *Not at All Likely*, 100 = *Very Likely*. Sex: 0 = male, 1 = female. Age (*M* = 18.71) and Self-Other Overlap (*M* = 3.45) in this model were mean-centered.

The second step of the hierarchical multiple regression analysis added self-other overlap to the model, allowing for a test of the association between self-other overlap and likelihood of working primarily with PWD controlling for the effects of sex and age. This step of analysis suggested self-other overlap was a significant predictor of likelihood of working primarily with PWD (*B* = 6.75, *β* = .375, *p* < .001), with this overall model predicting 15% of the variance in likelihood of primarily working with people with disability (*R*^*2*^ = .152). Sex remained a significant predictor (*B* = 5.27, *β* = .089, *p* = .018) of likelihood of working primarily with PWD. Additionally, age was a significant predictor (*B* = 1.69, *β* = .076, *p* = .042) of likelihood of working primarily with PWD, with each increasing year of age being associated with an increase in the reported likelihood of working primarily with PWD.

### Study 1 discussion

Study 1 was an initial test of the association between self-other overlap and willingness to work with PWD. As predicted, self-other overlap was significantly, and uniquely, positively associated with both openness to working with PWD, and likelihood of working primarily with PWD, as part of one’s future career. The findings of this study provided support for the overall study hypothesis by demonstrating an association between self-other overlap and willingness to work with people with disability in a sample of college undergraduates. In both hierarchical regression models, being female was associated with higher levels of willingness to work with PWD. This finding is consistent with previous work suggesting women are more willing to help than men [47,48]. As a result, gender was included as a covariate in Study 2 and Study 3.

## Study 2

Study 2 was designed to replicate study 1 and provide a further test of the overall study hypothesis by evaluating whether self-other overlap is a unique predictor of willingness to work with PWD, above and beyond the effect of trait empathy and attitudes toward PWD. Empathy and attitudes were selected as the targets of this evaluation because they are both key factors that have commonly been the focus of previous studies investigating helping behaviors and actions towards PWD [4,15,38,49,50]. In this study, attitudes were operationalized as explicit thoughts about the nature of PWD, while empathy was operationalized as individuals’ emotional response to others. These two components (i.e., explicit evaluative thoughts, and emotional responsiveness to others) were predicted to capture psychological aspects associated with willingness to work with people with disability that are separate from the sense of closeness and inclusion of the other in the self that was assessed through ratings of self-other overlap. Thus, based on work by Cialdini et al. [25] and Crisp and Turner [51], it was hypothesized that self-other overlap would be a significant unique positive predictor of willingness to work with PWD in models including self-other overlap, empathy, and attitudes.

### Materials and methods

#### Procedure

Participants signed-up for and completed an online survey through an undergraduate research pool portal. After agreeing to an electronic informed consent, they completed a battery of questions (96 total items) capturing their: level of self-other overlap with people with disability, state of empathy towards PWD, willingness to work with PWD, extent and diversity of previous experiences with PWD, feelings of similarity to PWD, trait empathy, attitudes toward PWD, and demographic information. At the conclusion of the survey, participants were thanked for their participation, awarded credit, and dismissed from the study. This study was approved by the Syracuse University Institutional Review Board.

#### Participants

Participants were undergraduates recruited from the psychology department participant pool. For quality assurance, participants were excluded from analyses if they completed the study in less than 4 minutes or greater than 115 minutes.

An a priori power analysis was conducted using G*Power 3.1. F tests were specified as the test family, and the statistical test was specified as linear multiple regression (fixed model, R^2^ deviation from zero). Based on the results of study 1 and the assumption that adding more variables to the model will not decrease the amount of variance explained by the overall model, the input parameters were: R^2^ = .15 (effect size f^2^ = .17647), α-error probability = .05, Power (1 –β-error probability) = .80, and total number of predictors = 12. This analysis determined 110 participants would provide sufficient power (power = 0.80) to detect the effects of a model with an overall R^2^ of .15.

#### Measures

**Self-other overlap, openness to working with people with disability, & likelihood of working primarily with people with disability.** The same items utilized in study 1 were used in this study.

**Anticipated percentage of time spent working with people with disability.** To capture an additional measure of willingness to work with PWD, participants were asked to report the percentage of their time they anticipate spending working with PWD as part of their future career (i.e., *When you are working in your future career*, *what percentage of your time will be spent working with people with disability*?*[Please enter a number from 0–100%*.*]*). They responded to this question by entering a value from 0–100%. This item was added starting with this study to provide a more concrete assessment of the amount to time individuals plan on investing in working with PWD as part of their career.

**Diversity and frequency of contact with people with disability.** Diversity and frequency of contact with PWD were assessed using a version of the Contact with Disabled Persons Scale [52] that was modified to focus on PWD in general (S1 Appendix). This scale includes 20 items (e.g., *How often have you had a long talk with a person who is disabled*?, *How often have you been annoyed or disturbed by the behavior of a person with a disability*?; Cronbach’s α = .92). Participants responded to these items by selecting one of 5 response options (0 = *Never*, 1 = *Once or twice*, 2 = *A few times*, 3 = *Often*, 4 = *Very often*). Even though some of these items refer to negative contact with PWD, they are not reverse-coded. The goal of the measure is simply to obtain an overall assessment of extent and diversity of contact [52]. A frequency of contact with PWD score was calculated for each participant by taking an average of participants’ ratings across all 20 items. A diversity of contact with PWD score was created by first recoding the responses to the 20 items such that 0 was still 0, but all other values above were coded as a 1. This allowed for a score to be created indicating the total number of experiences participants indicated having at least once or twice. These items were added to this study to provide a test of the conceptual validity of the self-other overlap measure. From a theoretical perspective, diversity and frequency of contact contribute to a portion of ones’ perception of self-other overlap.

**Trait empathy**. Trait empathy was assessed using the Toronto Empathy Questionnaire [53]. The questionnaire consists of 16 items (e.g., *when someone else is feeling excited*, *I tend to get excited too; It upsets me to see someone being treated disrespectfully*; *I get a strong urge to help when I see someone who is upset*), with half of the items reverse scored (e.g., *I do not feel sympathy for people who cause their own serious illnesses*; *I am not really interested in how other people feel*; *I find it silly for people to cry out of happiness;* Cronbach’s α = .90). Participants responded to these questions by selecting one of 5 response options (0 = *Never*, 1 = *Rarely*, 2 = *Sometimes*, 3 = *Often*, 4 = *Always*)

**State empathy toward people with disability.** To measure state empathy, participants were asked to rate the extent to which they experienced 6 emotions when they think about PWD (i.e., *Please indicate the extent to which you experience each of the following emotions when thinking about people with disability*: *sympathetic*, *soft-hearted*, *warm*, *compassionate*, *tender*, *moved;* Cronbach’s α = .95). Participants responded to this item using a 7-point scale (0 = *Not at All*; 6 = *Extremely*). A mean state empathy score was created for participants based on their responses to the 6 items. This state empathy measure was based on one used in similar previous studies [25,46,54–56].

**Attitudes toward people with disability.** Attitudes toward PWD was assessed using the Scale of Attitudes Towards Disabled Persons [57–59]. This instrument includes 24 items (e.g., *most disabled people are willing to work*, *disabled people show a deviant personality profile*, *disabled individuals can be expected to fit into competitive society;* Cronbach’s α = .84). Participants responded to these items using a 6-point scale (0 = *I disagree very much*, 1 = *I disagree pretty much*, 2 = *I disagree a little*, 3 = *I agree a little*, 4 = *I agree pretty much*, 5 = *I agree very much*). Half of the items were reverse coded.

**Similarity.** Participants were asked to respond to a single item to rate the extent to which they are similar to PWD (i.e., *How similar are you to people with disability*?). Participants responded to this item using a 7-point scale (0 = *Not Similar at All*; 6 = *Very Similar*). This item was based on previous work evaluating the association between self-other overlap and prosocial behavior for both individuals [32,54] and groups [60]. This item was added to this study to provide a test of the conceptual validity of the self-other overlap measure. From a theoretical perspective, feelings of similarity contribute to a portion of ones’ perception of self-other overlap.

**We-ness evaluation.** Participants were asked to indicate the extent to which they would use the term *we* to describe their relationship with PWD (i.e., *Please indicate to what extent you would use the term “we” to characterize you and people with disability*.). Participants responded to this item using a 7-point scale (0 = *Not at All*; 6 = *Extremely*). This item was based on one used by Cialdini et al. [25,46]. This item was added to this study to provide a test of the conceptual validity of the self-other overlap measure. From a theoretical perspective, ones’ sense of we-ness contributes to a portion of ones’ perception of self-other overlap.

**Disability attitude object definition.** Participants were asked to describe about whom they were thinking when responding to questions about PWD (i.e., *Describe the people you have been thinking about when you have been responding to the last several questions about people with disability*.). They provided their response to this open-ended question using a blank text box (S2 Appendix provides a selection of responses).

**Social desirability check.** The Marlowe-Crown Social Desirability Scale (Short Version–Form C) [61] was administered to participants at the end of the survey to provide a check for social desirability. This version had 13 items (e.g., *It is sometimes hard for me to go on with my work if I am not encouraged*; *No matter who I’m talking to*, *I’m always a good listener*), and participants responded to these items by indicating whether the statement was true (1) or false (0) with respect to themselves. Social desirability scores were created by calculating the sum of all 13 items for each participant (eight items were reverse coded). Higher scores indicated higher levels of social desirability.

**Age.** Age was assessed by allowing participants to type a number into a textbox.

**Level in college.** Level in college was assessed by allowing participants to select one of 4 options (0 = *Freshman*, 1 = *Sophomore*, 2 = *Junior*, 3 = *Senior*).

**Gender.** Studies 2 & 3 captured self-identified gender as a demographic variable instead of biological sex. Gender was assessed by allowing participants to select either man (0) or woman (1).

### Results

#### Descriptive statistics

There were a total of 177 participants utilized for analyses. One-hundred and eighty participants initiated the study, but 2 were excluded from analyses for completing the study in less than 4 minutes and 1 was eliminated for taking longer than 115 minutes to complete the study. The analysis sample was 81.4% freshman (n = 144; 11.9% sophomore, 4.5% junior, and 2.3% senior) and 68.9% identified as women (n = 122), with ages ranging from 18–33 (M = 18.95, SD = 1.45). Based on the obtained sample size and the a priori power calculations, this study was deemed to be sufficiently powered to test for the hypothesized associations.

Table 5 provides the means, standard deviations, and median values for the study variables. Both the mean (*M* = 3.32, *SD* = 1.69) and median (3) values for self-other overlap fell below the mid-point of the 7-point scale (1 = two non-overlapping circles, 7 = two almost completely overlapping circles). The mean ratings for primarily working with PWD (*M* = 40.82, *SD* = 28.71) and percentage of time anticipated working with PWD (*M =* 28.49, *SD* = 25.43) fell below the mid-point of the scale. The mean rating of openness to working with PWD (*M =* 67.44, *SD* = 27.40) fell above the mid-point of the scale. The mean ratings for trait empathy (*M =* 2.97, *SD* = .53) and attitudes (*M =* 3.39, *SD* = .63) were both also above the mid-point of the scale.

**Table 5:** Descriptive statistics for Study 2 variables.

	M	SD	Median
Self-Other Overlap	3.32	1.69	3
Openness	67.44	27.40	71
Primary	40.82	28.71	35
% Time	28.49	25.43	25
In-group/We-ness	2.80	2.12	3
Similarity	2.60	1.69	3
State Empathy	4.72	1.18	4.83
Trait Empathy (TEQ)	2.97	.53	3.00
Attitudes (SADP)	3.39	.63	3.37
Diversity of Contact	14.04	4.37	14
Frequency of Contact	1.44	.71	1.30
Social Desirability	5.79	2.74	6

*Note*. Self-Other Overlap: 1 = no overlap, 7 = highest level of overlap. Openness: 0 = Not at All Open, 100 = Very Open. Primary: 0 = Not at All Likely, 100 = Very Likely. % Time: 0–100%. In-group/We-ness: 0 = Not at All, 6 = Extremely. Similarity: 0 = Not Similar at All, 6 = Very Similar. State Empathy: 0 = Not at All, 6 = Extremely, averaged across 6 items. Trait Empathy: 0 = Never, 4 = Always, averaged across 16 items. Attitudes: 0 = I disagree very much, 5 = I agree very much, averaged across 24 items. Diversity of Contact: 0 = No, 1 = Yes, summed across 20 items. Frequency of Contact: 0 = Never, 4 = Very often, averaged across 20 items. Social Desirability: 0 = False, 1 = True, summed across 13 items.

#### Bivariate correlations

Table 6 provides the bivariate correlations for the study variables. As predicted, there was a positive association between self-other overlap and openness to working PWD (*r* = .156, *p* = < .05), primarily working with PWD (*r* = .220, *p* = < .01), and percentage of anticipated time working with PWD (*r* = .245, *p* = < .01). Additionally, self-other overlap was positively associated with diversity of contact (*r* = .253, *p* = < .01), frequency of contact (*r* = .345, *p* = < .01), we-ness (*r* = .393, *p* = < .01), and similarity (*r* = .254, *p* = < .01), supporting the conceptual validity of this construct. Given the magnitude of the correlations between these variables and self-other overlap, and their theoretical relationship, only self-other overlap was included in further analyses. Additionally, given the magnitude of the correlation between state empathy and trait empathy (*r* = .429, *p* = < .01), and that trait empathy is both associated with a higher number of willingness variables and more strongly associated with those variables (see Table 6), only trait empathy was included in further analyses. College level and social desirability were not significantly associated with the willingness variables, so they were also excluded from the further analyses.

**Table 6:** Study 2 bivariate correlations.

	SOO	Openness	Primary	% Time	State Empathy	Trait Empathy	Attitudes	Div. of Contact	Freq. of Contact	We-ness	Similarity	Age	College Level	Gender
Openness	.156*	1												
Primary	.220**	.577**	1											
% Time	.245**	.433**	.581**	1										
State Empathy	.139	.183*	.138	.109	1									
Trait Empathy (TEQ)	.173*	.251**	.131	.270**	.429**	1								
Attitudes	.161*	.364**	.130	.282**	.181*	.491**	1							
Div. of Contact	.253**	.126	.142	.250**	.139	-.022	.011	1						
Freq. of Contact	.345**	.284**	.323**	.381**	.297**	.188*	.265**	.790**	1					
We-ness	.393**	.047	.201**	.099	.191*	.151	-.002	.149	.226**	1				
Similarity	.254**	.221**	.319**	.241**	.077	.123	.114	.253**	.367**	.411**	1			
Age	.127	.110	.121	-.009	-.083	-.005	.075	.075	.133	.070	.087	1		
College Level	.047	.120	.138	-.005	-.145	.009	.017	.059	.058	.049	.095	.655**	1	
Gender	.034	.136	.104	.288**	.303**	.476**	.226**	-.032	.129	-.013	.074	-.125	-.108	1
Social Desirability	.012	.061	.095	.104	.065	.199*	.135	-.145	-.096	.100	.074	-.021	.003	.048

Note

* = Correlation is significant at the 0.05 level (2-tailed).

** = Correlation is significant at the 0.01 level (2-tailed).

#### Self-other overlap as a unique predictor of willingness to work with PWD

To assess whether self-other overlap is a unique predictor of the willingness variables above and beyond attitudes toward PWD and trait empathy, hierarchical multiple regression analyses were performed. For these analyses, self-other overlap was entered in the first step of the model. In the second, third, and fourth steps, attitudes toward PWD, trait empathy, and gender, respectively, were added to the analysis. In the model predicting openness to working with PWD (see Table 7), only attitudes was a significant predictor, with the overall model accounting for 15% of the variance in openness when attitudes was added in step 2 (R^2^ = .151). In the model predicting primarily working with PWD (see Table 8), only self-other overlap was a significant predictor, with the overall model accounting for 5% of the variance in primarily working with PWD when it was added in step 1 (R^2^ = .051), and the total variance explained not substantially changing with the addition of attitudes toward people with disability and trait empathy by the end of step 4 (R^2^ = .065). Finally, in the model predicting anticipated percent of time working with PWD (see Table 9), self-other overlap was the only significant predictor throughout all the steps it was included in the model. As with the model predicting primarily working with PWD, self-other overlap accounted for 5% of the variance in anticipated percent of time working with PWD when it was added in step 1 (R^2^ = .052). The final overall model including attitudes, trait empathy, and gender accounted for 15% of the variance in anticipated percent of time working with PWD (R^2^ = .150).

**Table 7:** Study 2 multiple regression analysis for self-other overlap, attitudes, and trait empathy predicting openness to working with people with disability.

	B	β	*t*	*p*	R^2^	*F*	df	*p*
Step 1					.020	2.86	1, 141	.093
Self-Other Overlap	2.23	.141	1.69	.093				
Constant	66.06							
Step 2					.151	12.41	2, 140	< .001
Self-Other Overlap	1.491	.094	1.20	.233				
Attitudes	15.73	.365	4.64	< .001				
Constant	12.66							
Step 3					.159	8.76	3, 139	< .001
Self-Other Overlap	1.35	.085	1.08	.283				
Attitudes	13.54	.314	3.51	.001				
Trait Empathy	5.37	.106	1.18	.240				
Constant	4.17							
Step 4					.159	6.54	4, 138	< .001
Self-Other Overlap	1.36	.086	1.08	.280				
Attitudes	13.56	.314	3.50	.001				
Trait Empathy	4.93	.097	.97	.332				
Gender	1.03	.018	.20	.842				
Constant	4.71							

*Note*. Openness to working with patients with disability: 0 = Not at All Open, 100 = Very Open. Gender: 0 = man, 1 = woman. Self-other Overlap in this model was median-centered (Median = 3). Attitudes (*M* = 3.39) and Trait Empathy (*M* = 2.97) in this model were mean-centered.

**Table 8:** Study 2 multiple regression analysis for self-other overlap, attitudes, and trait empathy predicting primarily working with people with disability.

	B	β	*t*	*p*	R^2^	*F*	df	*p*
Step 1					.051	7.25	1, 136	.008
Self-Other Overlap	3.87	.225	2.69	.008				
Constant	38.90							
Step 2					.063	4.51	2, 135	.013
Self-Other Overlap	3.65	.212	2.53	.013				
Attitudes	5.21	.110	1.32	.190				
Constant	21.21							
Step 3					.064	3.05	3, 134	.031
Self-Other Overlap	3.59	.209	2.47	.015				
Attitudes	4.31	.091	.96	.337				
Trait Empathy	2.33	.041	.44	.664				
Constant	17.36							
Step 4					.065	2.31	4, 133	.061
Self-Other Overlap	3.610	.210	2.48	.015				
Attitudes	4.36	.093	.97	.333				
Trait Empathy	1.40	.025	.24	.814				
Gender	2.13	.034	.36	.722				
Constant	18.48							

*Note*. Primarily working with patients with disability: 0 = Not at All Likely, 100 = Very Likely. Gender: 0 = man, 1 = woman. Self-other Overlap in this model was median-centered (Median = 3). Attitudes (*M* = 3.39) and Trait Empathy (*M* = 2.97) in this model were mean-centered.

**Table 9:** Study 2 multiple regression analysis for self-other overlap, attitudes, and trait empathy predicting anticipated percent time working with people with disability.

	B	β	*t*	*p*	R^2^	*F*	df	*p*
Step 1					.052	7.86	1, 142	.006
Self-Other Overlap	3.44	.229	2.80	.006				
Constant	26.89							
Step 2					.109	8.62	2, 141	< .001
Self-Other Overlap	2.93	.195	2.42	.017				
Attitudes	9.80	.240	2.99	.003				
Constant	-6.29							
Step 3					.130	6.96	3, 140	< .001
Self-Other Overlap	2.72	.181	2.26	.025				
Attitudes	6.55	.161	1.77	.079				
Trait Empathy	8.06	.166	1.83	.069				
Constant	-19.16							
Step 4					.150	6.92	4, 139	< .001
Self-Other Overlap	2.80	.186	2.34	.020				
Attitudes	6.59	.162	1.79	.075				
Trait Empathy	4.34	.090	.90	.370				
Gender	8.80	.160	1.80	.074				
Constant	-14.25							

*Note*. Anticipated percent time working with patients with disability: 0–100%. Gender: 0 = man, 1 = woman. Self-other Overlap in this model was median-centered (Median = 3). Attitudes (*M* = 3.39) and Trait Empathy (*M* = 2.97) in this model were mean-centered.

### Study 2 discussion

Study 1 demonstrated that self-other overlap does predict a significant amount of variance in variables related to willingness to work with PWD as assessed by measuring openness to working with people with disability and likelihood of working with people with disability. Study 2 built off this work by attempting to replicate those findings and evaluate self-other overlap as a unique predictor of willingness to work with PWD in models including self-other overlap, trait empathy, and attitudes. The findings from Study 2 replicated the findings of Study 1 with respect to self-other overlap being associated with openness to working with people with disability and likelihood of working with people with disability. Additionally, self-other overlap was associated with an additional willingness variable utilized in this study: to the percent of anticipated time spent working with people with disability. The findings from Study 2 also suggest that self-other overlap, overall, is a unique predictor of willingness to work with PWD in models including self-other overlap, trait empathy, and attitudes. There was an exception to this in that self-other overlap was not a significant unique predictor of openness to work with PWD. However, the findings of this study largely provide further support for the overall study hypothesis: self-other overlap is a unique, positive predictor of willingness to work with PWD as part of individuals’ future career.

## Study 3

Study 3 was developed to provide a further test of the overall study hypothesis and a conceptual replication of the findings of studies 1 and 2 by examining self-other overlap as a predictor of the groups of people participants indicate they would choose to help with their future work. Operationalizing willingness to work with PWD in this way provided an opportunity to evaluate whether self-other overlap is associated with the expression of a behavior directed toward PWD.

Specifically, for this study, participants were told that they would be participating in a study helping local non-profit organizations understand the psychological profiles of the people who support their causes so that these organizations can more effectively recruit people to work for them. After completing a skill inventory, participants were told they have skills that would be of value to these organizations and asked to honestly complete a series of questions about themselves and how they feel towards other groups of people. It was predicted that self-other overlap would be significantly positively associated with both the groups of people whom they selected as being most interested in working to help, and negatively associated with the groups whom they selected as being least interested in working to help. It was also predicted that self-other overlap would be a significant unique predictor of the groups whom were selected by the participants in models including trait empathy and attitudes.

### Materials and methods

#### Procedure overview

Participants completed this study online after signing-up through the psychology department’s undergraduate research pool portal. During the initiation of this study, participants were told they would be helping local non-profit organizations understand the psychological profiles of the people who support their causes so that these organizations can more effectively recruit people to work for them. After completing a skill inventory, participants were told they have skills that would be of value to these organizations and asked to honestly complete a series of questions about themselves and how they feel towards other groups of people. Participants were then asked to select the three groups of people whom they would most be interested in working with if they were recruited to work at a non-profit organization serving one of those groups. They were also asked to select the three groups of people whom they would be least interested in working with under the same conditions. After they made their selections, participants completed a series of self-other overlap items with different groups as the evaluation target, in addition to measures of trait empathy and attitudes towards PWD. The order of selecting the groups whom they are most and least interested in working with and the target groups of the measures were randomized to counteract any order effects. At the conclusion of the study, participants were thanked for their participation, awarded credit, and dismissed from the study. This study was reviewed and approved by Syracuse University Institutional Review Board.

#### Participants

Participants were undergraduates recruited from the psychology department participant pool. Participants were excluded from analyses if they completed the study in less than 4 minutes or greater than 115 minutes.

An a priori power analyses was conducted using G*Power 3.1. Z tests were specified as the test family, and the statistical test was specified as Logistic Regression. The input parameters were: Odds ratio = 1.5 (representing a small effect size of approximate .2 Cohen’s d; [62]), Pr(Y = 1|X = 1) H0 = .3 (representing the probably of selecting the group based on chance), α-error probability = .05, Power (1 –β-error probability) = .80, R2 other X = 0, X distribution = Normal, X parm μ = 0, and X parm σ = 1. This analysis determined 190 participants would provide enough power (power = 0.80) to detect the anticipated effects.

**Target groups.** There were 10 different target groups: 1. people with disability, 2. people who are homeless, 3. people who live in low-income housing, 4. people living with HIV, 5. people who are military veterans, 6. people who are refugees, 7. women who have been sexually assaulted, 8. people who are gay or lesbian, 9. people who want to start a business, and 10. people who are older adults.

**Groups selected with which to work.** Participants were asked to select three groups from a list with whom they would most be interested in working if they were to work for a non-profit specializing in serving that group (i.e., *If a non-profit organization serving one of the groups of people below needed someone with your skillset*, *which groups of people would you be most interested in working to help*? *[Please select the 3 groups of people*.*]*). Participants were able to select from the 10 different target groups.

**Groups selected with which to not work.** Participants were asked to select three groups from a list with whom they would be least interested in working if they were to work for a non-profit specializing in serving that group (i.e., *If a non-profit organization serving one of the groups of people below needed someone with your skillset*, *which groups of people would you be least interested in working to help*? *[Please select the 3 groups of people*.*]*). Participants were able to select from the 10 different target groups.

**Self-Other overlap.** Self-other overlap with the target group was measured using the same type of modified Inclusion of Other in the Self Scale used in studies 1 and 2. There were 10 self-other overlap items, one for each group (i.e., *people with disability*, *people who are homeless*, *people who live in low-income housing*, *people living with HIV*, *people who are military veterans*, *people who are refugees*, *women who have been sexually assaulted*, *people who are gay or lesbian*, *people who want to start a business*, *and people who are older adults*.). Participants were asked to select the pair of circles that best represents their relationship with each target group.

**Trait empathy, attitudes toward people with disability, demographics.** The same items utilized in study 2 were used in this study.

### Results

A total of 229 participants completed with study, with 218 participants utilized in analyses. The included sample was 74.3% freshman (n = 162; 17.9% sophomore, 3.2% junior, and 3.7% senior) and 66.1% identified as women (n = 144), with ages ranging from 18–33 (M = 18.81, SD = 1.36). Eleven participants were excluded from analyses because of the duration quality check. One participant completed the study in less than 4 minutes (240 seconds), and 10 participants took longer than 115 minutes (6900 seconds) to complete the study. Based on the obtained sample size and the a priori power calculations, this study was deemed to be sufficiently powered to test for the hypothesized associations.

PWD (*M* = 3.30, *SD* = 1.73), people who are gay/lesbian (*M* = 3.57, *SD* = 1.82), people who want to start a business (*M* = 3.86, *SD* = 1.99), and people who are older adults (*M* = 4.44, *SD* = 1.64) were the four groups for whom participants provided the highest mean ratings of self-other overlap. People who are homeless (*M* = 2.18, *SD* = 1.47), people who are military veterans (*M* = 2.04, *SD* = 1.81), people who are refugees (*M* = 2.04, *SD* = 1.49), and people living with HIV (*M* = 1.59, *SD* = 1.28) were the four groups for whom participants provided the lowest mean ratings of self-other overlap. People who live in low-income housing (*M* = 2.85, *SD* = 1.66) and women who have been sexually assaulted (*M* = 2.93, *SD* = 1.87) were two groups whom were neither in the highest or lowest four based on mean rating of self-other overlap.

#### Bivariate correlations

Table 10 provides the bivariate correlations for the association between the study variables for each group and the selection outcomes (i.e., selecting a group as one most interested in working to help, or selecting a group as least interested in working to help). Self-other overlap was positively associated with selecting PWD as a group with whom they were most interested in working to help (MWH; *r* = .220, *p* = < .01), and it was negatively associated with selecting PWD as a group with whom they were least interested in working to help (LWH; *r* = -.136, *p* = < .05). This means that people who have high levels of self-other overlap with PWD were more likely to select PWD as a group with whom they are most interested in working to help, and less likely to select PWD as a group with whom they are least interested in working to help.

**Table 10:** Study 3 bivariate correlation values for the association between each study variable and the choice to either indicate a desire to work with or not work with a group of people.

	Dis.		H		LI		HIV		Vet.		Ref.		WSA		G/L		B		OA	
	MHW	LHW	MHW	LHW	MHW	LHW	MHW	LHW	MHW	LHW	MHW	LHW	MHW	LHW	MHW	LHW	MHW	LHW	MHW	LHW
Self-Other Overlap	.220**	-.136*	.179**	-.138*	.256**	-.059	.080	-.111	.305**	-.152*	.090	-.180**	.253**	-.068	.188**	-.264**	.433**	-.401**	.126	-.144*
Trait Empathy	.088	-.284**	.107	-.039	-.005	.035	-.002	.053	-.227**	-.009	.080	-.050	.172*	-.045	-.071	-.067	-.114	.127	-.134	.222**
Att. Toward PWD	.051	-.177*																		

*Note*: Dis. = people with disability, H = people who are homeless, LI = people who live in low-income housing, HIV = people living with HIV, Vet. = people who are military veterans, Ref. = people who are refugees, WSA = women who have been sexually assaulted, G/L = people who are gay or lesbian, B = people who want to start a business, OA = people who are older adults. MHW = Selecting group as most interested in working to help. LHW = Selecting group as least interested in working to help.

* = Correlation is significant at the 0.05 level (2-tailed).

** = Correlation is significant at the 0.01 level (2-tailed).

The same pattern of association (i.e., self-other overlap positively associated with MWH, and negatively associated with LWH) was also observed when the target groups were: people who are homeless (MWH: *r* = .179, *p* = < .01; LWH: *r* = -.138, *p* = < .05), people who are military veterans (MWH: *r* = .305, *p* = < .01; LWH: *r* = -.152, *p* = < .05), people who are gay/lesbian (MWH: *r* = .188, *p* = < .01; LWH: *r* = -.264, *p* = < .01), and people who want to start a business (MWH: *r* = .433, *p* = < .01; LWH: *r* = -.401, *p* = < .01). For 4 of the other 5 groups (i.e., people who live in low-income housing, people who are refugees, women who have experienced sexual assault, and people who are older adults), self-other overlap was only associated with one of the selection variables. People living with HIV was the only group for whom self-other overlap was not associated with one of the selection variables.

Table 11 provides bivariate correlations for each of the study variables related to PWD. Self-other overlap was the only predictor variable associated with selecting PWD as the group whom people were most interested in helping with their career (*r* = .220, *p* = < .01. However, trait empathy (*r* = -.284, *p* = < .01), attitudes toward people with disability (*r* = -.177, *p* = < .05), and college level (*r* = .188, *p* = < .01) were all associated with selecting PWD as the group whom people were least interested in helping with their career, in addition to self-other overlap (*r* = -.136, *p* = < .05).

**Table 11:** Study 3 bivariate correlations for variables related to people with disability.

	MWH	LWH	SOO	Trait Empathy	Att. Toward PWD	Age	College Level	Gender
LHW	-.383**	1						
SOO	.220**	-.136*	1					
Trait Empathy	.119	-.284**	.192**	1				
Att. Toward PWD	.051	-.177*	.172*	.586**	1			
Age	-.066	.048	.048	-.026	.034	1		
College Level	-.090	.188**	.005	-.123	.008	.686**	1	
Gender	.112	-.084	-.034	.234**	.218**	-.033	-.114	1

*Note*: MHW = Selecting group as most interested in working to help. LHW = Selecting group as least interested in working to help. SOO = Self-other overlap with people with disability.

* = Correlation is significant at the 0.05 level (2-tailed).

** = Correlation is significant at the 0.01 level (2-tailed).

#### Self-other overlap as a unique predictor of selecting people with disability

Multiple logistic regression analyses were conducted to test whether self-other overlap is a unique predictor of selecting PWD as either the group whom they would most be interested, or least interested, in working to help when accounting for attitudes toward PWD and trait empathy. In the hierarchical multiple logistic regression model predicting selecting PWD as a group whom participants were most interested in working to help (see Table 12), only self-other overlap was a significant predictor. The overall model accounted for 7.5% of the variance in selecting PWD (R^2^ = .075), with self-other overlap (OR = 1.32, *p* = .002) accounting for 5.3% of the variance when it was added in the first step (R^2^ = .053). In the hierarchical multiple logistic regression model predicting selecting PWD as a group participants are least interested in working to help (see Table 13), self-other overlap was not a significant predictor at any step in the model. The final overall model included self-other overlap, attitudes toward PWD, trait empathy, and gender, and accounted for 7.2% of the variance in selecting PWD (R^2^ = .072). Trait empathy was the only significant predictor of selecting PWD in the final step of the model (OR = 0.36, *p* = .028).

**Table 12:** Study 3 multiple logistic regression analysis for self-other overlap, attitudes, and trait empathy predicting selecting people with disability as a group participants were most interested in working to help.

	B	OR	*95% OR CI*	*p*	Cox & Snell R^2^	*Χ*^*2*^	df	*p*
Step 1					.053	9.98	1	.002
Self-Other Overlap	.278	1.32	1.11–1.58	.002				
Constant	-.355							
Step 2					.053	9.99	2	.007
Self-Other Overlap	.277	1.32	1.10–1.58	.002				
Attitudes	.027	1.03	0.58–1.82	.926				
Constant	-.355							
Step 3					.061	11.62	3	.009
Self-Other Overlap	.266	1.31	1.09–1.56	.004				
Attitudes	-.238	0.79	0.39–1.60	.508				
Trait Empathy	.538	1.71	0.74–3.96	.208				
Constant	-.348							
Step 4					.075	14.37	4	.006
Self-Other Overlap	.283	1.33	1.11–1.59	.003				
Attitudes	-.330	0.72	0.35–1.48	.370				
Trait Empathy	.475	1.61	0.69–3.77	.275				
Gender	.560	1.75	0.90–3.42	.101				
Constant	-.723							

*Note*. Selecting people with disability: 0 = No, 1 = Yes. Gender: 0 = man, 1 = woman. Self-other Overlap in this model was median-centered (Median = 3). Attitudes (*M* = 3.18) and Trait Empathy (*M* = 2.75) in this model were mean-centered.

**Table 13:** Study 3 multiple logistic regression analysis for self-other overlap, attitudes, and trait empathy predicting selecting people with disability as a group participants were least interested in working to help.

	B	OR	*95% OR CI*	*p*	Cox & Snell R^2^	*X*^*2*^	df	*p*
Step 1					.016	2.96	1	.086
Self-Other Overlap	-.174	0.84	0.69–1.03	.093				
Constant	-1.01							
Step 2					.043	8.15	2	.017
Self-Other Overlap	-.150	0.86	0.70–1.06	.158				
Attitudes	-.760	0.47	0.24–0.91	.026				
Constant	-1.05							
Step 3					.071	13.56	3	.004
Self-Other Overlap	-.131	0.88	0.71–1.08	.226				
Attitudes	-.201	0.82	0.36–1.85	.630				
Trait Empathy	-1.06	0.35	0.14–0.87	.025				
Constant	-1.09							
Step 4					.072	13.88	4	.008
Self-Other Overlap	-.136	0.87	0.71–1.08	.210				
Attitudes	-.172	0.84	0.37–1.92	.682				
Trait Empathy	-1.03	0.36	0.14–0.90	.028				
Gender	-.209	0.81	0.39–1.67	.569				
Constant	-.959							

*Note*. Selecting people with disability: 0 = No, 1 = Yes. Gender: 0 = man, 1 = woman. Self-other Overlap in this model was median-centered (Median = 3). Attitudes (*M* = 3.18) and Trait Empathy (*M* = 2.75) in this model were mean-centered.

### Study 3 discussion

Study 1 and study 2 provided evidence to suggest that self-other overlap is a unique predictor of willingness to work with PWD. Study 3 furthered the understanding of this association in three ways. First, this study demonstrated that self-other overlap was associated with choices people make when selecting groups with whom they would be interested in working to help. This is important because it suggests self-other overlap is not associated solely with items focusing on PWD. Rather, it is also associated with selecting PWD as a group with which to work to help in a context where disability is not the focus and a variety of other options are available. Second, this study suggests that self-other overlap is associated with willingness to work with other groups of people in addition to PWD. The findings indicate that self-other overlap may not predict willingness in the same way for all groups of people, but for almost every group in this study (people living with HIV was the only exception), self-other overlap was associated with participants’ choices related to using their work to help the group. Finally, this study suggests, that factors associated with selecting a group as one whom participants are most interested in working to help, and the factors associated with selecting a group as one whom participants are least interested in working to help may be different. For people with disability, this is demonstrated by self-other overlap being a unique predictor of selecting this group as one whom they are most interested in helping with their work. However, it was not a unique predictor of choosing not to work with PWD in models including trait empathy and attitudes toward PWD. Additional evidence for this conclusion can be found in the pattern of association between self-other overlap and selections made with respect to the other target groups in this study. Overall, these findings provide further support for the overall study hypothesis.

## General discussion

The goal of this set of studies was to evaluate whether self-other overlap is a unique predictor of willingness to work with PWD. Across three studies, evidence indicates that self-other overlap was uniquely associated with both multiple measures of willingness to work with PWD (studies 1 & 2), and selecting PWD as a group to help with one’s work (study 3). Thus, these findings suggest that self-other overlap is a unique predictor of willingness to work with PWD, supporting the overall study hypothesis.

### Placing the findings in context

The findings of this work make several key theoretical contributions. First, ratings of self-other overlap were shown, again, to be associated with choices related to long-term, high-investment helping behavior. It has long been argued that prosocial behavior is driven by selfless, altruistic motives [54,56,63–65]. However, accumulating evidence suggests both short-term and long-term helping behavior may be driven by more self-focused motives (i.e., helping the people with whom one has the highest level of self-other overlap) [6,8,9,17,19,20,22,25]. The results of this study provide another instance where ratings of self-other overlap are associated with long-term, high investment helping behavior (e.g., choosing to primarily work with PWD, choosing to spend a high percentage of time working with PWD). However, it should be noted that both empathy and attitudes, at times, were also unique predictors of willingness to work with PWD. This suggests that all three of these factors, (i.e., inclusion of the other in the self, empathy, and attitudes) may contribute to these prosocial career decisions.

Second, finding that ratings of self-other overlap are uniquely associated with the groups that individuals select with whom to work highlights the potential role selfish motives may play in seemingly prosocial career decisions. This is important for several key reasons. First, this work suggests that, if given the choice, people might use their work to help those to whom they feel closest and see as being most like themselves. Recognizing this helps to identify a potential mechanism that could underlie the preferential treatment of one group of people over another in work-related contexts (i.e., maximizing benefits to clients, selecting mentees, allowing exceptions to company policies). Additionally, this work provides insight into a potential issue that can undermine the efforts of professional recruitment pipelines that have been put in place to benefit underserved populations or high need areas. Regardless of people’s background, if they do not feel a sense of closeness and connection with the underserved population, they may be less likely to use their career to help them.

Finally, many disability-focused interventions have implicitly acknowledged the importance of influencing ones’ sense of self-other overlap to create long-term change, but it has not been explicitly identified as a potentially unique target for intervention. This is reflected in the rationale laid out for the interventions that include components aimed at developing empathy or providing service providers with experiences interacting with PWD [38,66]. The authors of these interventions discuss the importance of the service providers developing a sense of closeness with people with disability, but it is often mentioned in the context of altering attitudes or empathy [5,36,38]. PWD themselves extol the importance of service providers “seeing” beyond their disability and connecting with them as individuals [67,68], but, again, the solutions proposed often focus on changing attitudes or increasing empathy [38,39,69,70]. This work suggests that the sense of closeness and integration associated with seeing members of a group as overlapping with oneself is a factor that is quantitatively different from thinking positively about a group or feeling empathy for people. As such, it may be a useful target for intervention.

### Limitations and future directions

One of the primary limitations of this work is that that none of the studies have the dimension of temporal precedence necessary to test whether participants’ sense of self-other overlap influences participants’ willingness to work with PWD. Studies 1 and 2 asked participants to provide their ratings of self-other overlap before they were asked to answer items related to their willingness to work with PWD, while study 3 asked participants to make their group selections before self-other overlap was measured, and the association between the variables remained consistent. Reversing the order in which participants completed this measure helps to address concerns about the timing of the self-other overlap item biasing the responses to the willingness items. However, the battery of questions in all three of the studies was asked in the same moment in time. Given that the goal of this work was to take the initial steps to explore whether a relationship exists between self-other overlap and willingness to work with PWD, its correlational nature was appropriate. However, it does mean that more work will be needed to understand the dynamics of any causal relationship that might exist.

Another key limitation to this work is that the willingness measures have not been validated. The novelty of the items and the associated lack of validation makes it hard to know whether these items capture a true measure of thoughts that would naturally occur as people are making career decisions. It is likely that most people will never explicitly ask themselves the percent of their work time that they plan on spending serving a group of people. However, it may be possible that they will ask themselves whether they are open to working with a particular group of people, or if they are willing to commit to working primarily with a group of people (i.e., teachers choosing to work in an inner-city school district). Thus, there is a level of face-validity to these items and they are all strongly associated with one another, but the differences in means and level of investment and commitment between the individual items suggest more work is needed to further assess their validity.

In a related vein, even if the willingness measures are found to have high levels of construct validity, it is not known whether responses to these items are associated with the groups of people with whom individuals ultimately work as part of their career. There are many different factors that impact the work people ultimately do to make money and support themselves. In many cases, these decisions are driven by social, relational, fiscal, and other individual, interpersonal, and systems-level factors that have very little connection to the feelings individuals have towards the group of people they are serving through their work. Thus, it seems likely that self-other overlap and the responses participants provide on items capturing the groups whom they are willing to serve with their work will be most predictive in circumstances where individuals have a high level of autonomy to make choices about the work they do (i.e., medical students choosing a medical specialty, people selecting an organization at which to volunteer). Therefore, even though these studies create a context where all of the participants are able to indicate their willingness related to their future work, it is possible that the connection between their willingness and their actual work, if one exists, may only be strong for a subset of participants. Again, more work is needed to better understand how thoughts related to work with particular groups of people translates into career decisions and work behavior.

In the future, if it is demonstrated that self-other overlap does have an impact on willingness to work with PWD and the career decisions people make with respect to work with this portion of the population, more work will be needed to identify effective, efficient intervention components. Previous work has demonstrated that self-other overlap is malleable in experimental contexts [23,24,71–74]. However, it is likely current educational intervention activities, when considered from the perspective of self-other overlap, include elements that would enhance feelings of closeness between participants and PWD. This includes activities that help people draw connections between themselves and PWD, and provide opportunities to gain experience (e.g., perspective taking activities, facilitating informal interpersonal interactions, and having multiple interactions in different contexts over time). These kinds of activities provide a starting point for designing interventions that facilitate self-other overlap with PWD.

### Conclusion

Overall, the findings from this work suggest that ratings of self-other overlap are uniquely associated with willingness to work with people with disability as part of one’s career. More research is needed to understand whether willingness translates into people serving PWD through their work, and to explore whether interventions designed to increase self-other overlap do increase willingness to work with PWD. However, this work does suggest the sense of closeness and connection people feel toward PWD may be important to take into consideration when developing interventions targeted toward promoting working with PWD.

## Supporting information

S1 Appendix Contact with Disabled Persons Scale. (DOCX)

S2 Appendix Disability attitude object definition open-ended responses. (DOCX)

S1 Survey Survey questions for Study 1. (DOCX)

S2 Survey Survey questions for Study 2. (DOCX)

S3 Survey Survey questions for Study 3. (DOCX)

S1 Dataset Study 1 Dataset. (XLSX)

S2 Dataset Study 2 Dataset. (XLSX)

S3 Dataset Study 3 Dataset. (XLSX)
