# Supplementary material for: Self-other overlap: A unique predictor of willingness to work with people with disability as part of one’s career
Source: PLoS One. 2019 Aug 12;14(8):e0220722. doi: 10.1371/journal.pone.0220722 (PMC6690537; doi:10.1371/journal.pone.0220722)
Supplement: S1 Appendix — (DOCX) [file pone.0220722.s001.docx]

Supporting Information

**S1 Appendix**

Contact with Disabled Persons Scale

1. How often have you had a long talk with a person who is disabled?
2. How often have you had brief conversations with persons who are disabled?
3. How often have you eaten a meal with a person who is disabled?
4. How often have you contributed money to organization that help disabled persons?
5. How often have disabled persons discussed their lives or problems with you?
6. How often have you discussed your life or problems with a disabled person?
7. How often have you tried to help disabled persons with their problems?
8. How often have disabled persons tried to help you with your problems?
9. How often have you worked with a disabled client, student, or patient on the job?
10. How often have you worked with a disabled co-worker?
11. How often has a disabled friend visited you in your home?
12. How often have you visited disabled friends in their homes?
13. How often have you met a disabled person that you like?
14. How often have you met a disabled person that you dislike?
15. How often have you met a disabled person that you admire?
16. How often have you met a disabled person for whom you feel sorry?
17. How often have you been annoyed or disturbed by the behavior of a person with a disability?
18. How often have you been pleased by the behavior of a disabled person?
19. How often have you had pleasant experiences interacting with disabled persons?
20. How often have you had unpleasant experiences interacting with disabled persons?

Never = 0, Once or twice = 1, A few times = 2, Often = 3, Very often = 4

This scale is modified to focus on people with disability in general. The original, unmodified scale is presented in

Yuker HE, Hurley MK. Contact with and attitudes toward persons with disabilities: The measurement of intergroup contact. Rehabil Psychol. 1987;32: 145–154. doi:10.1037/h0091569
