# Supplementary material for: Self-other overlap: A unique predictor of willingness to work with people with disability as part of one’s career
Source: PLoS One. 2019 Aug 12;14(8):e0220722. doi: 10.1371/journal.pone.0220722 (PMC6690537; doi:10.1371/journal.pone.0220722)
Supplement: S2 Appendix — (DOCX) [file pone.0220722.s002.docx]

Supporting Information

**S2 Appendix**

Disability Attitude Object Definition Open-Ended Responses (Verbatim)

| People with disabilities | I have been thinking about people with a mental disability |
| --- | --- |
| I think of all the experiences I have had disabled people are the most genuine and kindest people I have known and they are more than competent in a number of different ways and often have a way of seeing things other people cannot. | When i was in high school I would volunteer in a room with disabled children, about 5-10 of them. For an hour a day, 5 days a week i would go and play games with them and talk to them |
| Nobody in specific, maybe kids with special needs in certain schools | Anything from people with speech disabilities, learning disabilities, to people with physical defects |
| My cousin with a mild form of autism, and because my mom is a nurse I have gone and helped her take care of tons of young and old individuals who have types of disabilities and it is very eye opening | I have been thinking of people with physical disabilities such as missing limbs, blindness, deafness, and other types of disabilities such as down syndrome and autism |
| My friends cousin. | People I have gone to school with, people I have seen. |
| people with physical and mental disabilities | The people I have been thinking about are those with autism or down syndrome |
| I have been thinking about people who can't walk, can't see (blind) and those who speak sign language since i have had personal contact and spoken to people who speak sign language | I have been thinking about a number of individuals such as those with mental illnesses like Down Syndrome and Autism and also physically handicap individuals like those paralyzed. |
| People with autism, down syndrome, etc. | People in wheelchairs, people with cognitive abilities |
| I have been thinking about people I've seen at my school and some distant friends siblings who have disabilities, down syndrome comes to mind first for me | I think of my cousin and I think he is one of the smartest people I have ever met, he just lacks in social cues. I find that to be true of most disabled people, so I have a big heart for them. |
| People with disabilities are just people. There is nothing that makes them different its something that makes them special | People with cerebral palsy, down syndrome, learning disabilities, disabilities due to injury, and any kind of disability one can have. |
| My neighbor, and people I have been with while volunteering. | People who have down syndrome, people who are not able to walk, hear or see. |
| Those who possess disabilities such as down syndrome | My cousin who has an autism. |
| I have been thinking about people that work in YMCA and also people in my parish | SPECIAL NEEDS KIDS at my high school with Autism |
| my friends brother great kid | about an good experience i had with a disabled person |
| I have been thinking about people with a mental disability. | a friend of mine who is completely paralyzed due to living through a war |
| I have mainly been thinking about my friend's little brother with down syndrome and my boyfriend's little brother with autism. | A friend I know with spinea bifiada (can't spell but it prevents him from walking/having full motion control) My sister and people I know with severe learning disabilities, OCD, ADHA |
| People in Wheelchairs, my friends with Autism or severe ADHD | They are always happy, in their own world. As long as I see them being happy and enjoying life, then I do not have to feel sorry. |
| Disabled students I met at school and disabled adults I have oberved/occasionally interacted with in public settings | Most of them are within my family, but I have also been thinking about children and other adults who I have either come into contact with or worked with. |
| My basketball coaches daughter is disabled and is very independent and kind-hearted. She loves the movie pitch-perfect and is overall a warm person. | I have been thinking about my cousin as he has gone through many therapists, the expenses my aunt has had to find the money for, the time she devotes to make sure he has what he needs, etc. He is decently high functioning but the littles of things can set him off. He is quite smart but the most simple tasks he has trouble grasping sometimes, he also is grade levels behind but has trouble being in a mainstreamed environment. He is the kindest most sweetest boy. He always tries to make someone smile and can know when you're upset. I hope one day he can have a steady job and grasp simple concepts, I know he can do it. |
| The people I have met with disabilities struggle but have shown their warmth and happiness as well. I have also met those who are unable to perform jobs properly, cannot speak, or act out in a way that tells me they aren't receiving proper attention. |  |
| Usually small children or older people with disabilities. |  |
| 1. my brother had a stroke when he was younger and had trouble reading and writing, and also temperament issues; gets angry very easily  2. disabled people that work at my local store  3. disabled person that my coach has brought to our softball practice a few times | The people I have met with disabilities struggle but have shown their warmth and happiness as well. I have also met those who are unable to perform jobs properly, cannot speak, or act out in a way that tells me they aren't receiving proper attention. |
| Motivational speaker that came to speak to all the kids at a lacrosse event. Kids at my high school who were in several of my classes / friends with at school. | I have been thinking about my friends brother with MS and my assigned buddy when i volunteer who is on the spectrum. |
| My uncle, and friend with a disability I knew in high school. | I envisioned a mental disability, such as a learning disorder |
| I have been thinking of my high school special education students. | My friend from high school who has a mental disability and my distant cousin. |
| There are so many different disabilities that affect people in differing ways and degrees that it is impossible to generalize "disability" as one. | I have been thinking about my special needs campers I worked with over the summer as well as people I know with disabilities such as ADHD and OCD |
| I believe people with disability are able to live a life similar to mine, but they may need a little extra help | I have been thinking about my cousin who has downs and my friend's younger brother who has autism. |
| People with a mental or physical disability that can sometimes prevent them from functionally "normally" in life | I've been thinking about my best friend's brother, who is 17 years old and has a complicated and unknown disability. |
| people with learning disabilities or mental problems | I have been thinking of my roommate and my brother. |
| My mom's friend's son is disabled with autism | My aunt who is physically disabled |
| My cousin My brother in law My neighbors at home | my sister has adhd and sometimes acts out and doesn't pay attention but she is smart and capable of doing anything she wants to |
| Depends. Both people with mental and physical disabilities. | I have been thinking about my cousin and one of my former neighbors. |
| Homeless people | Kids from my town who have various disabilities |
| My brother was born prematurely with a disability. | I was thinking about my mother who has positively impacted my life the most. |
| I know a neighbor who has a disability, and she is very kind and competent is some manners. | I was thinking about my own experiences with people with disabilities during Best Buddies |
| The elderly people in nursing homes that I've interacted with. | disabled kids in my high school, and town, and my second cousin |
| I have been thinking about my older brother a lot while responding to these last several questions with regards to disabilities. | The person from my last college who is blind, my coworker at my last job, severely autistic people I have met while volunteering |
| My mother, who has a physical disability, and other people who have mental disabilities | People that have a disability but are still able to work and function on their own in a productive manner. |
| I have been thinking about my cousin. He has autism and, though it is severe, has been making great progress. I have also been thinking about a girl I went to high school with who was often incredibly rude to others, whether she meant to be or not, with a learning disability and mild autism. | The people that I have been thinking about are often people who volunteer with me at the hospital who are disabled and I have seen just how capable they are. And also a lot of the children who were in my group during my summer camp with whom I interacted a lot with. |
| When I think of disability, I think of people who may have disabilities with their body (like injured), people in wheel chairs, people with mental disabilities, and people with disorders like Down Syndrome. | I thought of people with mental disabilities as those are the kinds of kids with disabilities I've worked with i the past and was what first came to mind. I also took into consideration people with physically disabilities |
| Sometimes I thought about the children who had autism that I worked with, but I also thought about people who were perhaps missing an arm or leg. | I have been thinking about the incredibly strong and inspirational people I have learned about through reading autobiographies. |
| For the most part I have been thinking of mental disability, but for some I thought physical. | People with physical abilities, such as down syndrome or in a wheelchair. |
| I've been thinking about a couple of students from my High School who had down syndrome, and I got to know each of them pretty well. | People with any disability that has problems with being around people or just not getting along well with people are who I am thinking about. |
| Both myself and my close friends with hearing loss, but also those with learning disabilities that I have interacted with over the years. | I have thought about people with mental disabilities such as something as minor as ADHD. Also, those who are physically disabled. |
| People I know with disabilities or my friends and my experiences I've had with them. | My aunt, and disabled people that I know within my community. |
| Happy, usually smiling, always wants to talk and hug. | People in wheelchairs |
| childhood friend. neighbor. | My friend's sister in general. |
| I was familiar with many children that had down syndrome in my high school | That people with disabilities have a stereotype around them that they are bad people |
