# Supplementary material for: Self-other overlap: A unique predictor of willingness to work with people with disability as part of one’s career
Source: PLoS One. 2019 Aug 12;14(8):e0220722. doi: 10.1371/journal.pone.0220722 (PMC6690537; doi:10.1371/journal.pone.0220722)
Supplement: S1 Survey — (DOCX) [file pone.0220722.s003.docx]

**Study 1 Survey Questions**

1) Please select the pair of circles that best represents your relationship with people with disability. 
[S = Self, PWD = People with Disability]


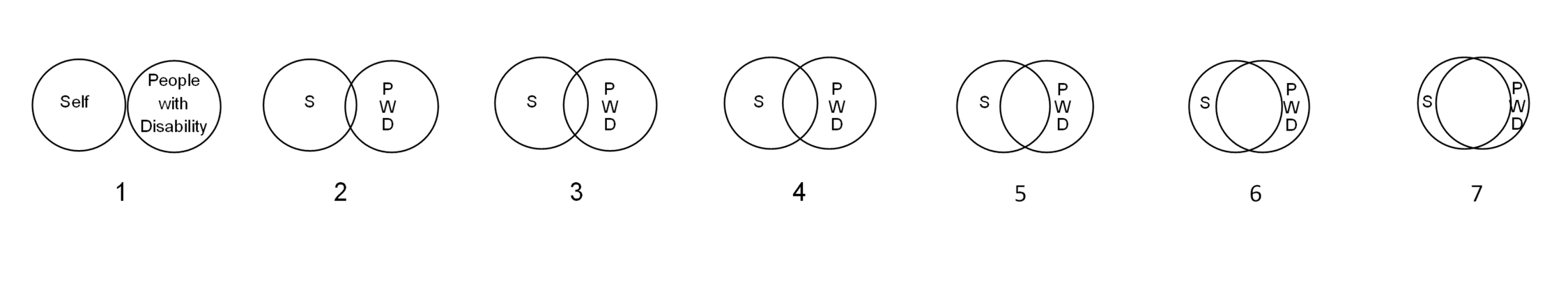


2) Please use the slider to indicate the extent to which you are open to working with people with disability as part of your future career.

|  | Not at All Open | Very Open |
| --- | --- | --- |

|  | 0 | 10 | 20 | 30 | 40 | 50 | 60 | 70 | 80 | 90 | 100 |
| --- | --- | --- | --- | --- | --- | --- | --- | --- | --- | --- | --- |

| (1) | 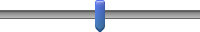 |
| --- | --- |

3) Please use the slider to indicate the extent to which you are likely to work primarily with people with disability as part of your future career.

|  | Not at All Likely | Very Likely |
| --- | --- | --- |

|  | 0 | 10 | 20 | 30 | 40 | 50 | 60 | 70 | 80 | 90 | 100 |
| --- | --- | --- | --- | --- | --- | --- | --- | --- | --- | --- | --- |

| (1) | 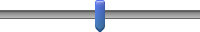 |
| --- | --- |

4) Age: ______

5) Level in College:

1. Freshman

2. Sophomore

3. Junior

4. Senior

5. Other. Please specify: _______________________________________

6) Sex:

Male

Female
