# Supplementary material for: Self-other overlap: A unique predictor of willingness to work with people with disability as part of one’s career
Source: PLoS One. 2019 Aug 12;14(8):e0220722. doi: 10.1371/journal.pone.0220722 (PMC6690537; doi:10.1371/journal.pone.0220722)
Supplement: S2 Survey — (DOCX) [file pone.0220722.s004.docx]

**Study 2 Survey Questions**

1) Please select the pair of circles that best represents your relationship with people with disability. [S = Self, PWD = People with Disability]


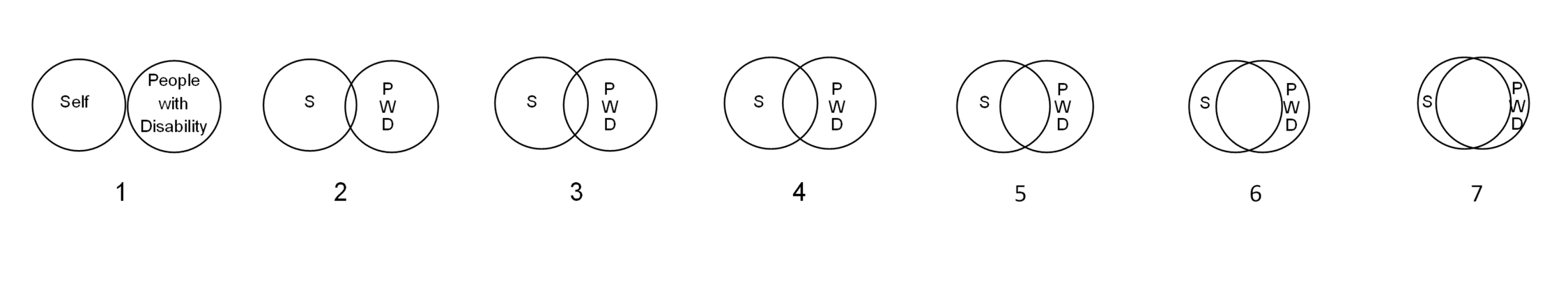


2) Please indicate to what extent you would use the term "we" to characterize you and people with disability.

- Not at All (1)
- (2)
- (3)
- (4)
- (5)
- (6)
- Extremely (7)

3-8) Please indicate the extent to which you experience each of the following emotions when thinking about people with disability:

|  | Not at All (1) | (2) | (3) | (4) | (5) | (6) | Extremely (7) |
| --- | --- | --- | --- | --- | --- | --- | --- |
| Sympathetic (1) |  |  |  |  |  |  |  |
| Soft-hearted (2) |  |  |  |  |  |  |  |
| Warm (3) |  |  |  |  |  |  |  |
| Compassionate (4) |  |  |  |  |  |  |  |
| Tender (5) |  |  |  |  |  |  |  |
| Moved (6) |  |  |  |  |  |  |  |

7) Please use the slider to indicate the extent to which you are open to working with people with disability as part of your future career.

|  | Not at All Open | Very Open |
| --- | --- | --- |

|  | 0 | 10 | 20 | 30 | 40 | 50 | 60 | 70 | 80 | 90 | 100 |
| --- | --- | --- | --- | --- | --- | --- | --- | --- | --- | --- | --- |

| (1) | 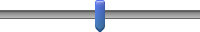 |
| --- | --- |

8) Please use the slider to indicate the extent to which you are likely to work primarily with people with disability as part of your future career.

|  | Not at All Likely | Very Likely |
| --- | --- | --- |

|  | 0 | 10 | 20 | 30 | 40 | 50 | 60 | 70 | 80 | 90 | 100 |
| --- | --- | --- | --- | --- | --- | --- | --- | --- | --- | --- | --- |

| (1) | 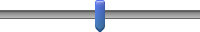 |
| --- | --- |

9) When you are working in your future career, what percentage (%) of your time will be spent working with people with disability?  [Please enter a number from 0-100.]

10-29) Please select a response option to indicate your answer to each question.

|  | Never (1) | Once or twice (2) | A few times (3) | Often (4) | Very often (5) |
| --- | --- | --- | --- | --- | --- |
| How often have you had a long talk with a person who is disabled? (1) |  |  |  |  |  |
| How often have you had brief conversations with persons who are disabled? (2) |  |  |  |  |  |
| How often have you eaten a meal with a person who is disabled? (3) |  |  |  |  |  |
| How often have you contributed money to organization that help disabled persons? (4) |  |  |  |  |  |
| How often have disabled persons discussed their lives or problems with you? (5) |  |  |  |  |  |
| How often have you discussed your life or problems with a disabled person? (6) |  |  |  |  |  |
| How often have you tried to help disabled persons with their problems? (7) |  |  |  |  |  |
| How often have disabled persons tried to help you with your problems? (8) |  |  |  |  |  |
| How often have you worked with a disabled client, student, or patient on the job? (9) |  |  |  |  |  |
| How often have you worked with a disabled co-worker? (10) |  |  |  |  |  |
| How often has a disabled friend visited you in your home? (11) |  |  |  |  |  |
| How often have you visited disabled friends in their homes? (12) |  |  |  |  |  |
| How often have you met a disabled person that you like? (13) |  |  |  |  |  |
| How often have you met a disabled person that you dislike? (14) |  |  |  |  |  |
| How often have you met a disabled person that you admire? (15) |  |  |  |  |  |
| How often have you met a disabled person for whom you feel sorry? (16) |  |  |  |  |  |
| How often have you been annoyed or disturbed by the behavior of a person with a disability? (17) |  |  |  |  |  |
| How often have you been pleased by the behavior of a disabled person? (18) |  |  |  |  |  |
| How often have you had pleasant experiences interacting with disabled persons? (19) |  |  |  |  |  |
| How often have you had unpleasant experiences interacting with disabled persons? (20) |  |  |  |  |  |

30) How similar are you to people with disability?

- Not Similar at All (1)
- (2)
- (3)
- (4)
- (5)
- (6)
- Very Similar (7)

31-37) Please select a response option to indicate how well each statement describes yourself.

|  | Does not Describe Me Very Well (1) | Very well (2) | Moderately well (3) | Describes Me Very Well (4) |
| --- | --- | --- | --- | --- |
| I often have tender, concerned feelings for people less fortunate than me. (1) |  |  |  |  |
| Sometimes I don't feel very sorry for other people when they are having problems. (2) |  |  |  |  |
| When I see someone being taken advantage of, I feel kind of protective towards them. (3) |  |  |  |  |
| Other people's misfortunes do not usually disturb me a great deal. (4) |  |  |  |  |
| When I see someone being treated unfairly, I sometimes don't feel very much pity for them. (5) |  |  |  |  |
| I am often quite touched by things that I see happen. (6) |  |  |  |  |
| I would describe myself as a pretty soft-hearted person. (7) |  |  |  |  |

38-53) Please read each statement carefully and rate how frequently you feel or act in the manner described. There are no right or wrong answers or trick questions. Please answer each question as honestly as you can.

|  | Never (1) | Rarely (2) | Sometimes (3) | Often (4) | Always (5) |
| --- | --- | --- | --- | --- | --- |
| When someone else is feeling excited, I tend to get excited too. (1) |  |  |  |  |  |
| Other people’s misfortunes do not disturb me a great deal. (2) |  |  |  |  |  |
| It upsets me to see someone being treated disrespectfully. (3) |  |  |  |  |  |
| I remain unaffected when someone close to me is happy. (4) |  |  |  |  |  |
| I enjoy making other people feel better. (5) |  |  |  |  |  |
| I have tender, concerned feelings for people less fortunate than me. (6) |  |  |  |  |  |
| When a friend starts to talk about his\her problems, I try to steer the conversation towards something else. (7) |  |  |  |  |  |
| I can tell when others are sad even when they do not say anything. (8) |  |  |  |  |  |
| I find that I am “in tune” with other people’s moods. (9) |  |  |  |  |  |
| I do not feel sympathy for people who cause their own serious illnesses. (10) |  |  |  |  |  |
| I become irritated when someone cries. (11) |  |  |  |  |  |
| I am not really interested in how other people feel. (12) |  |  |  |  |  |
| I get a strong urge to help when I see someone who is upset. (13) |  |  |  |  |  |
| When I see someone being treated unfairly, I do not feel very much pity for them. (14) |  |  |  |  |  |
| I find it silly for people to cry out of happiness. (15) |  |  |  |  |  |
| When I see someone being taken advantage of, I feel kind of protective towards him\her. (16) |  |  |  |  |  |

54-77) The statements presented below express opinions or ideas about people who are disabled. There are many differences of opinion; Many people agree and many people disagree with each statement. We would like to know your opinion about them. Select the response option which best corresponds with how you feel about the statement. There are not right or wrong answers You should work as quickly as you can, but don’t rush. There is no time limit. Please respond to every statement.

|  | I disagree very much (1) | I disagree pretty much (2) | I disagree a little (3) | I agree a little (4) | I agree pretty much (5) | I agree very much (6) |
| --- | --- | --- | --- | --- | --- | --- |
| Disabled children should not be provided with a free public education. (1) |  |  |  |  |  |  |
| Disabled people are not more accident prone than other people. (2) |  |  |  |  |  |  |
| A disabled individual is not capable of making moral decisions. (3) |  |  |  |  |  |  |
| Disabled people should be prevented from having children. (4) |  |  |  |  |  |  |
| Disabled people should be allowed to live where and how they choose. (5) |  |  |  |  |  |  |
| Adequate housing for disabled people is neither too expensive nor too difficult to build. (6) |  |  |  |  |  |  |
| Rehabilitation programs for disabled people are too expensive to operate. (7) |  |  |  |  |  |  |
| Disabled people are in many ways like children. (8) |  |  |  |  |  |  |
| Disabled people need only the proper environment an opportunity to develop and express criminal tendencies. (9) |  |  |  |  |  |  |
| Disabled adults should be involuntarily committed to an institution following arrest. (10) |  |  |  |  |  |  |
| Most disabled people are willing to work. (11) |  |  |  |  |  |  |
| Disabled individuals are able to adjust to a life outside an institutional setting. (12) |  |  |  |  |  |  |
| Disabled people should not be prohibited from obtaining a driver’s license. (13) |  |  |  |  |  |  |
| Disabled people should live with others of similar disability. (14) |  |  |  |  |  |  |
| Zoning ordinances should not discriminate against disabled people by prohibiting group homes in residential districts. (15) |  |  |  |  |  |  |
| The opportunity for gainful employment should be provided to disabled people. (16) |  |  |  |  |  |  |
| Disabled children in regular classrooms have an adverse effect on other children. (17) |  |  |  |  |  |  |
| Simple repetitive work is appropriate for disabled people. (18) |  |  |  |  |  |  |
| Disabled people show a deviant personality profile. (19) |  |  |  |  |  |  |
| Equal employment opportunities should be available to disabled individuals. (20) |  |  |  |  |  |  |
| Laws to prevent employers from discriminating against disabled people should be passed. (21) |  |  |  |  |  |  |
| Disabled people engage in bizarre and deviant sexual activity. (22) |  |  |  |  |  |  |
| Disabled workers should receive at least the minimum wage establishes for their jobs. (23) |  |  |  |  |  |  |
| Disabled individuals can be expected to fit into competitive society. (24) |  |  |  |  |  |  |

78) People with disability are warm.

- Strongly disagree (1)
- (2)
- (3)
- (4)
- (5)
- (6)
- Strongly agree (7)

79) People with disability are competent.

- Strongly disagree (1)
- (2)
- (3)
- (4)
- (5)
- (6)
- Strongly agree (7)

80) Describe the people you have been thinking about when you have been responding to the last several questions about people with disability.

________________________________________________________________

________________________________________________________________

81-93) Listed below are a number of statements concerning personal attitudes and traits. Read each item and decide whether the statement is true or false as it pertains to you.

|  | True (1) | False (0) |
| --- | --- | --- |
| It is sometimes hard for me to go on with my work if I am not encouraged. (1) |  |  |
| I sometimes feel resentful when I don’t get my way. (2) |  |  |
| On a few occasions, I have given up doing something because I thought too little of my ability. (3) |  |  |
| There have been times when I felt like rebelling against people in authority even though I knew they were right. (4) |  |  |
| No matter who I’m talking to, I’m always a good listener. (5) |  |  |
| There have been occasions when I took advantage of someone. (6) |  |  |
| I’m always willing to admit it when I make a mistake. (7) |  |  |
| I sometimes try to get even rather than forgive and forget. (8) |  |  |
| I am always courteous, even to people who are disagreeable. (9) |  |  |
| I have never been irked when people expressed ideas very different from my own. (10) |  |  |
| There have been times when I was quite jealous of the good fortune of others. (11) |  |  |
| I am sometimes irritated by people who ask favors of me. (12) |  |  |
| I have never deliberately said something that hurt someone’s feelings. (13) |  |  |

94) Level in College

- Freshman (1)
- Sophomore (2)
- Junior (3)
- Senior (4)
- Other (please specify) (5) ________________________________________________

95) Gender

- Man (0)
- Woman (1)
- Self-Identify (-99) ________________________________________________

96) Age

________________________________________________________________
