# Supplementary material for: Self-other overlap: A unique predictor of willingness to work with people with disability as part of one’s career
Source: PLoS One. 2019 Aug 12;14(8):e0220722. doi: 10.1371/journal.pone.0220722 (PMC6690537; doi:10.1371/journal.pone.0220722)
Supplement: S3 Survey — (DOCX) [file pone.0220722.s005.docx]

**Study 3 Survey Questions**

Skills Inventory

Our lab is working with local non-profit organizations to develop general trait profiles of people who may be interested in working with the populations that they serve. This information will be used to help them better target their marketing and talent recruitment efforts.

To start-off, we are going to have you complete an initial skill inventory to see if you have a skill set that might be of interest to our partners.

Page Break

1-25) Please consider whether or not you possess each of the skills listed below. Please check the "yes" and "no" options to the right of each skill depending on whether or not you believe yourself to possess the skill in question.

|  | Yes (1) | No (4) |
| --- | --- | --- |
| setting work/committee goals (1) |  |  |
| defining performance standards (2) |  |  |
| managing people (3) |  |  |
| motivating others (4) |  |  |
| expressing feelings (5) |  |  |
| setting priorities (6) |  |  |
| conducting interviews (7) |  |  |
| delegating responsibilities (8) |  |  |
| running meetings (9) |  |  |
| writing letters/papers/proposals (10) |  |  |
| reading volumes of material (11) |  |  |
| sketching charts or diagrams (12) |  |  |
| taking personal responsibility (13) |  |  |
| managing an organization (14) |  |  |
| persuading others (15) |  |  |
| creating meaningful and challenging work (16) |  |  |
| comparing results (17) |  |  |
| mediating between people (18) |  |  |
| enforcing rules and regulations (19) |  |  |
| dispensing information (20) |  |  |
| budgeting expenses (21) |  |  |
| raising funds (22) |  |  |
| interviewing prospective employees (23) |  |  |
| calculating numerical data (24) |  |  |
| encouraging others (25) |  |  |

Page Break

Thank you! You have skills that our non-profit partners would value.

We are now going to ask you a series of questions about yourself and your feelings towards other groups of people. We know that sometimes people respond to these questions in ways that do not reflect how they truly feel because they are concerned about what other people might think of their responses. However, for us to best help these organizations, we need you to answer these questions as honestly as possible.

The survey you are completing is completely anonymous. This means that nobody will be able to connect you to any of the responses you provide.

Page Break

Choosing Groups

26) If a non-profit organization serving one of groups of people below needed someone with your skillset, which groups of people would you be *most* interested in working to help? [Please select 3 groups of people.]

- People with disability (1)
- People who are homeless (2)
- People who live in low-income housing (3)
- ​People living with HIV (4)
- ​People who are military veterans (5)
- People who are ​refugees (6)
- ​Women who have experienced sexual assault (7)
- ​People who are gay or lesbian (8)
- ​People who want to start a business (9)
- Older adults (10)

27) If a non-profit organization serving one of groups of people below needed someone with your skillset, which groups of people would you be *least* interested in working to help? [Please select 3 groups of people.]

- People with disability (1)
- People who are homeless (2)
- People who live in low-income housing (3)
- ​People living with HIV (4)
- ​People who are military veterans (5)
- People who are refugees (6)
- ​Women who have experienced sexual assault (7)
- ​People who are gay or lesbian (8)
- ​People who want to start a business (9)
- Older adults (10)

28) Please select the pair of circles that best represents your relationship with people with disability. [S = Self, PWD = People with Disability]

-
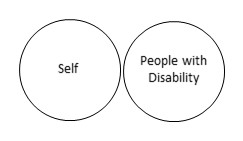
(1)
-
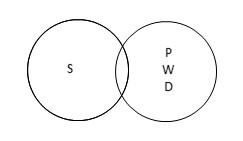
  (2)
-
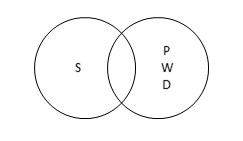
 (3)
-
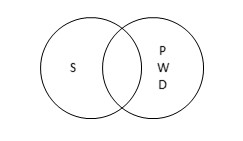
 (4)
-
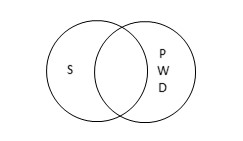
(5)
-
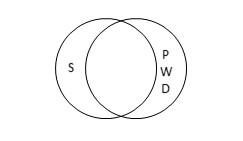
 (6)
-
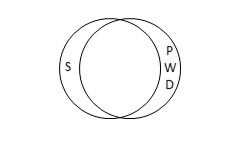
(7)

29) Please select the pair of circles that best represents your relationship with people who are homeless [S = Self, PWAH = People who are homeless]

-
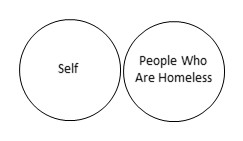
(1)
-
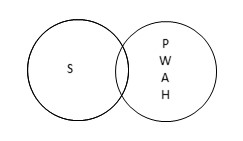
 (2)
-
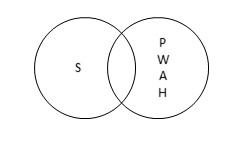
 (3)
-
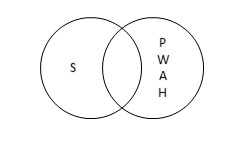
 (4)
-
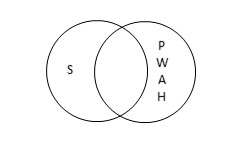
(5)
-
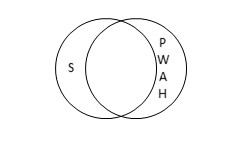
(6)
-
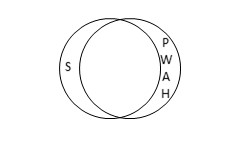
 (7)

30) Please select the pair of circles that best represents your relationship with people who live in low-income housing. [S = Self, PLIH=People who live in low-income housing]

-
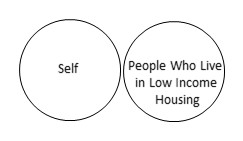
 (1)
-
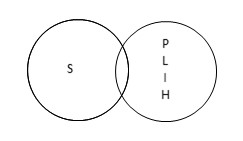
 (2)
-
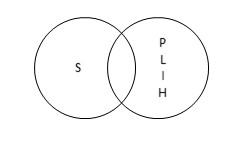
(3)
-
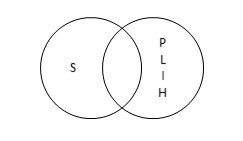
 (4)
-
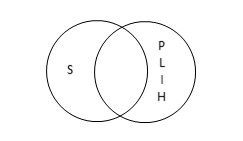
 (5)
-
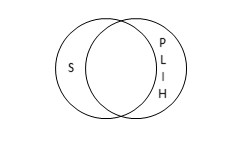
 (6)
-
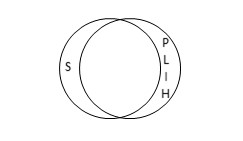
 (7)

31) Please select the pair of circles that best represents your relationship with people living with HIV. [S = Self, PHIV=People living with HIV]

-
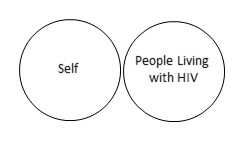
 (1)
-
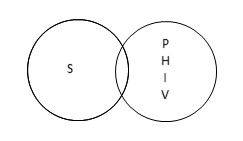
  (2)
-
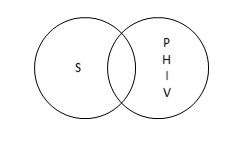
  (3)
-
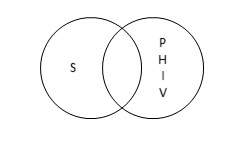
 (4)
-
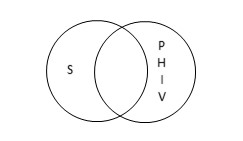
 (5)
-
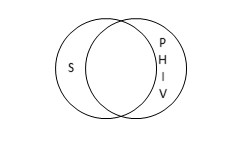
 (6)
-
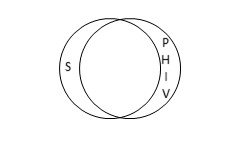
 (7)

32) Please select the pair of circles that best represents your relationship with people who are military veterans. [S = Self, MV=People who are military veterans]

-
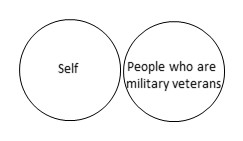
  (1)
-
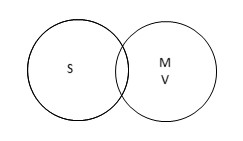
 (2)
-
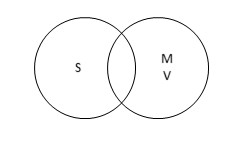
  (3)
-
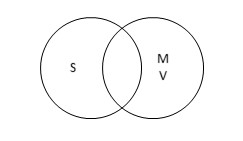
 (4)
-
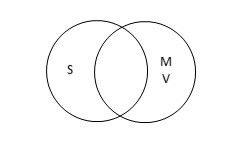
 (5)
-
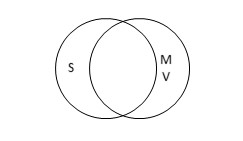
 (6)
-
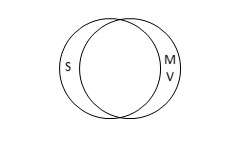
 (7)

33) Please select the pair of circles that best represents your relationship with people who are refugees. [S = Self, R = People who are refugees]

-
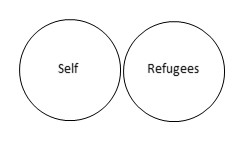
  (1)
-
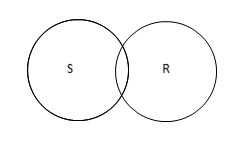
  (2)
-
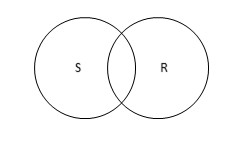
  (3)
-
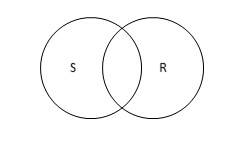
  (4)
-
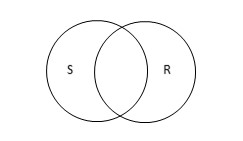
  (5)
-
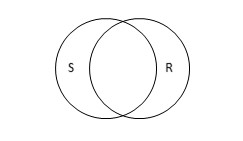
  (6)
-
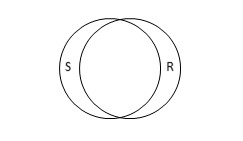
  (7)

34) Please select the pair of circles that best represents your relationship with women who have experienced sexual assault. [S = Self, WSA = Women who have experienced sexual assault]

-
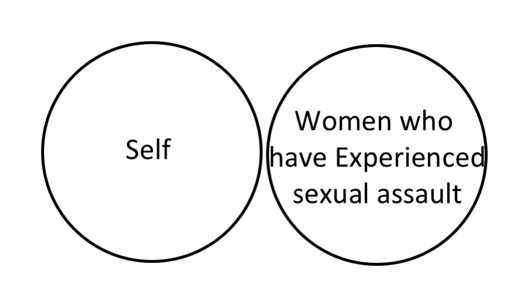
  (1)
-
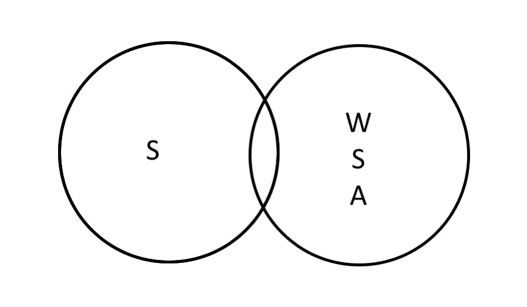
  (2)
-
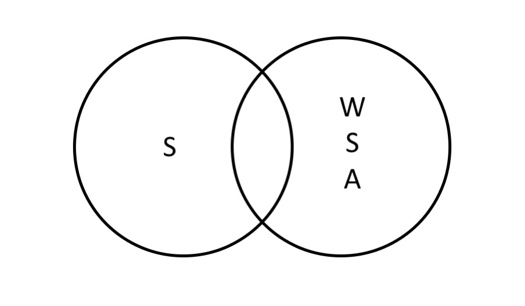
 (3)
-
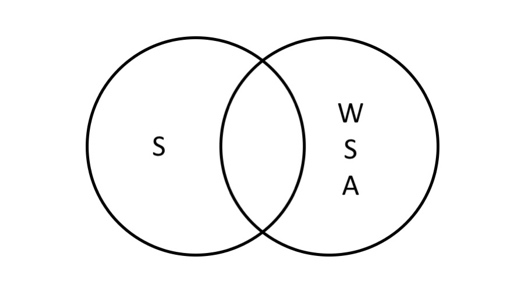
(4)
-
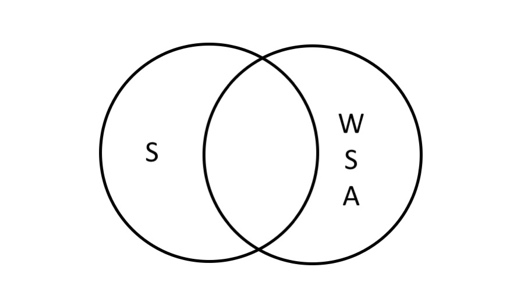
  (5)
-
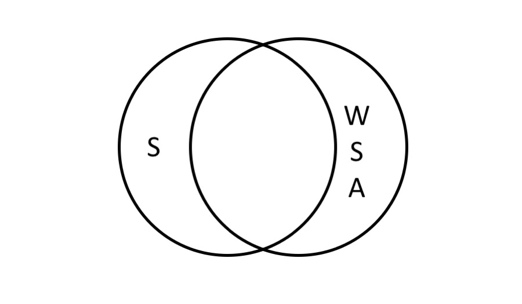
  (6)
-
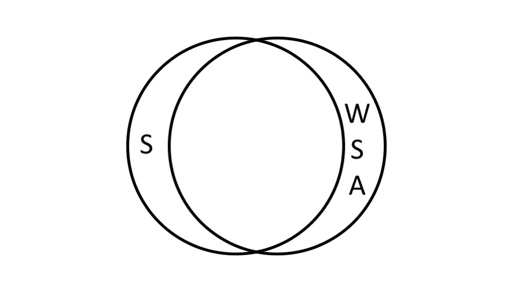
 (7)

35) Please select the pair of circles that best represents your relationship with people who are gay or lesbian. [S = Self, G&L=People who are gay or lesbian.]

-
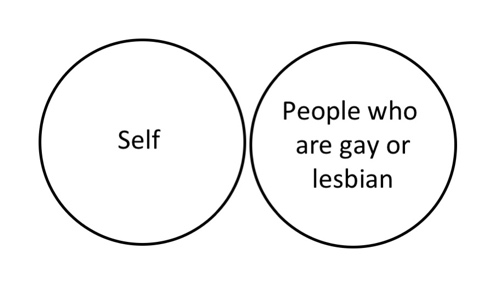
 (1)
-
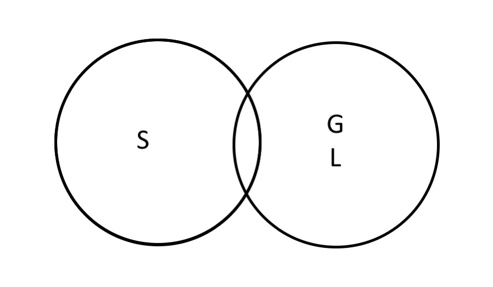
  (2)
-
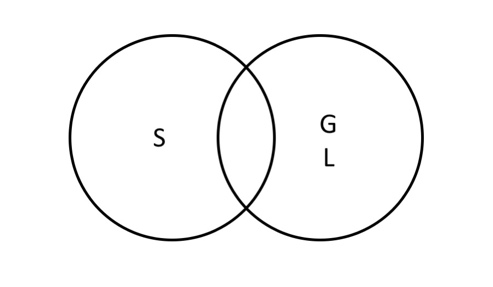
  (3)
-
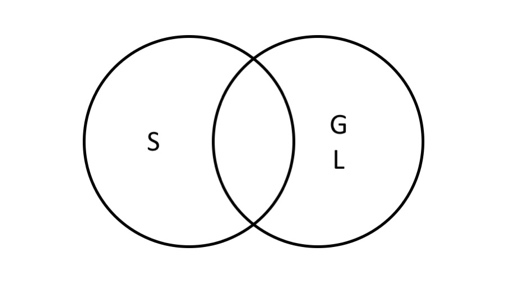
 (4)
-
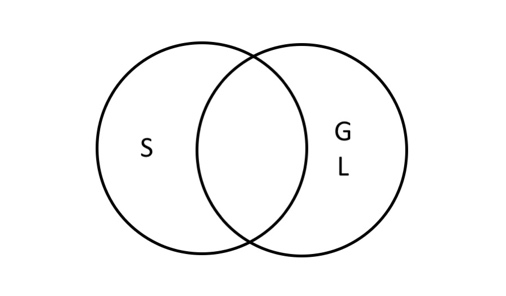
 (5)
-
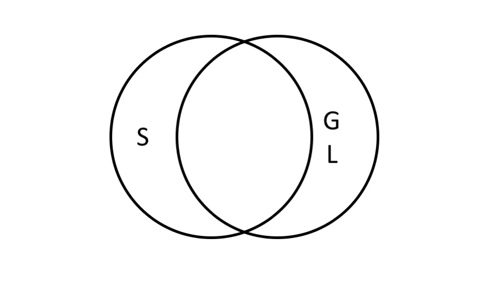
 (6)
-
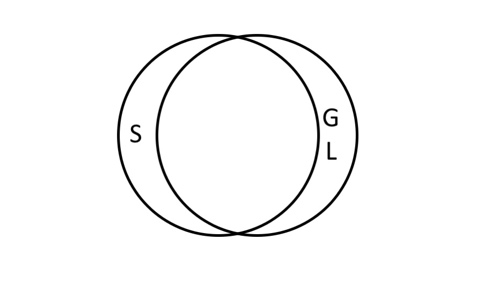
 (7)

36) Please select the pair of circles that best represents your relationship with people who want to start a business. [S = Self, E=Entrepreneurs.]

-
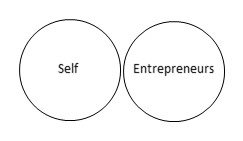
(1)
-
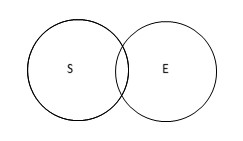
  (2)
-
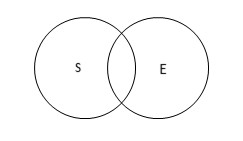
  (3)
-
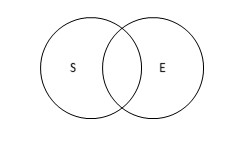
 (4)
-
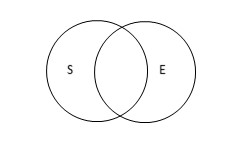
 (5)
-
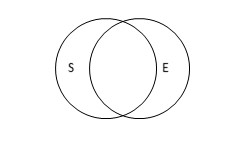
 (6)
-
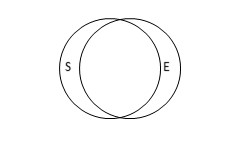
 (7)

37) Please select the pair of circles that best represents your relationship with older adults [S = Self, OA=Older adults.]

-
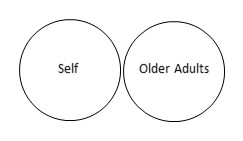
(1)
-
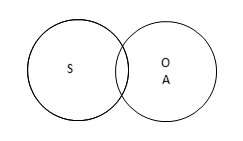
  (2)
-
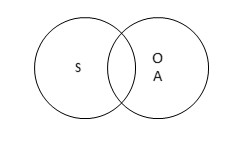
 (3)
-
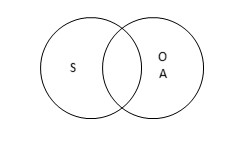
  (4)
-
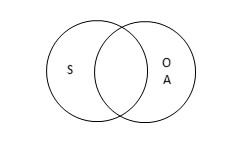
  (5)
-
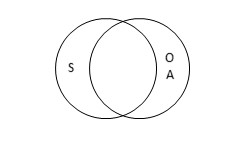
  (6)
-
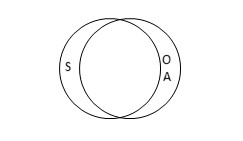
  (7)

38-53) Please read each statement carefully and rate how frequently you feel or act in the manner described. There are no right or wrong answers or trick questions. Please answer each question as honestly as you can.

|  | Never (1) | Rarely (2) | Sometimes (3) | Often (4) | Always (5) |
| --- | --- | --- | --- | --- | --- |
| When someone else is feeling excited, I tend to get excited too. (1) |  |  |  |  |  |
| Other people’s misfortunes do not disturb me a great deal. (2) |  |  |  |  |  |
| It upsets me to see someone being treated disrespectfully. (3) |  |  |  |  |  |
| I remain unaffected when someone close to me is happy. (4) |  |  |  |  |  |
| I enjoy making other people feel better. (5) |  |  |  |  |  |
| I have tender, concerned feelings for people less fortunate than me. (6) |  |  |  |  |  |
| When a friend starts to talk about his\her problems, I try to steer the conversation towards something else. (7) |  |  |  |  |  |
| I can tell when others are sad even when they do not say anything. (8) |  |  |  |  |  |
| I find that I am “in tune” with other people’s moods. (9) |  |  |  |  |  |
| I do not feel sympathy for people who cause their own serious illnesses. (10) |  |  |  |  |  |
| I become irritated when someone cries. (11) |  |  |  |  |  |
| I am not really interested in how other people feel. (12) |  |  |  |  |  |
| I get a strong urge to help when I see someone who is upset. (13) |  |  |  |  |  |
| When I see someone being treated unfairly, I do not feel very much pity for them. (14) |  |  |  |  |  |
| I find it silly for people to cry out of happiness. (15) |  |  |  |  |  |
| When I see someone being taken advantage of, I feel kind of protective towards him\her. (16) |  |  |  |  |  |

54-77) The statements presented below express opinions or ideas about people who are disabled. There are many differences of opinion; Many people agree and many people disagree with each statement. We would like to know your opinion about them. Select the response option which best corresponds with how you feel about the statement. There are not right or wrong answers You should work as quickly as you can, but don’t rush. There is no time limit. Please respond to every statement.

|  | I disagree very much (1) | I disagree pretty much (2) | I disagree a little (3) | I agree a little (4) | I agree pretty much (5) | I agree very much (6) |
| --- | --- | --- | --- | --- | --- | --- |
| Disabled children should not be provided with a free public education. (1) |  |  |  |  |  |  |
| Disabled people are not more accident prone than other people. (2) |  |  |  |  |  |  |
| A disabled individual is not capable of making moral decisions. (3) |  |  |  |  |  |  |
| Disabled people should be prevented from having children. (4) |  |  |  |  |  |  |
| Disabled people should be allowed to live where and how they choose. (5) |  |  |  |  |  |  |
| Adequate housing for disabled people is neither too expensive nor too difficult to build. (6) |  |  |  |  |  |  |
| Rehabilitation programs for disabled people are too expensive to operate. (7) |  |  |  |  |  |  |
| Disabled people are in many ways like children. (8) |  |  |  |  |  |  |
| Disabled people need only the proper environment an opportunity to develop and express criminal tendencies. (9) |  |  |  |  |  |  |
| Disabled adults should be involuntarily committed to an institution following arrest. (10) |  |  |  |  |  |  |
| Most disabled people are willing to work. (11) |  |  |  |  |  |  |
| Disabled individuals are able to adjust to a life outside an institutional setting. (12) |  |  |  |  |  |  |
| Disabled people should not be prohibited from obtaining a driver’s license. (13) |  |  |  |  |  |  |
| Disabled people should live with others of similar disability. (14) |  |  |  |  |  |  |
| Zoning ordinances should not discriminate against disabled people by prohibiting group homes in residential districts. (15) |  |  |  |  |  |  |
| The opportunity for gainful employment should be provided to disabled people. (16) |  |  |  |  |  |  |
| Disabled children in regular classrooms have an adverse effect on other children. (17) |  |  |  |  |  |  |
| Simple repetitive work is appropriate for disabled people. (18) |  |  |  |  |  |  |
| Disabled people show a deviant personality profile. (19) |  |  |  |  |  |  |
| Equal employment opportunities should be available to disabled individuals. (20) |  |  |  |  |  |  |
| Laws to prevent employers from discriminating against disabled people should be passed. (21) |  |  |  |  |  |  |
| Disabled people engage in bizarre and deviant sexual activity. (22) |  |  |  |  |  |  |
| Disabled workers should receive at least the minimum wage establishes for their jobs. (23) |  |  |  |  |  |  |
| Disabled individuals can be expected to fit into competitive society. (24) |  |  |  |  |  |  |

78) Level in College

- Freshman (1)
- Sophomore (2)
- Junior (3)
- Senior (4)
- Other (please specify) (5) ________________________________________________

79) Gender

- Man (0)
- Woman (1)
- Self-Identify (-99) ________________________________________________

80) Age

________________________________________________________________
